# Properties characterization and microstructural analysis of alkali-activated solid waste-based materials with sawdust and wastewater integration

**DOI:** 10.1371/journal.pone.0313413

**Published:** 2025-01-03

**Authors:** Liang Li, Xianhui Zhao, Haoyu Wang, Jianran Cao, Xian-en Zhao

**Affiliations:** 1 School of Civil Engineering, Tianjin Renai College, Tianjin, China; 2 School of Civil Engineering, Hebei University of Engineering, Handan, China; 3 Hebei Yingsheng New Material Technology Co., Ltd., Shijiazhuang, China; Mirpur University of Science and Technology, PAKISTAN

## Abstract

Construction materials are significantly exposed to ecological hazards due to the presence of hazardous chemical constituents found in industrial and agricultural solid wastes. This study aims to investigate the use of sawdust particles (SDPs) and sawdust wastewater (SDW) in alkali-activated composites (AACs) made from a mixture of different silicon-aluminum-based solid wastes (slag powder-SP, red mud-RM, fly ash-FA, and carbide slag-CS). The study examines the impact of SDP content, treated duration of SDPs, and SDW content on both fresh and hardened properties of the AACs, including electrical conductivity, fluidity, density, flexural and compressive strengths, and drying shrinkage. The study also analyzes the microstructures and product compositions of the AACs influenced by SDW through a comprehensive analysis of microstructures and product compositions by using XRD, SEM-EDS, and FTIR. The results show that treating SDPs with a 2.5 mol/L NaOH solution for 12 hours decreases the fluidity and electrical conductivity of the AACs but improves their flexural and compressive strengths. Additionally, in the synthesis of a composite material incorporating binder materials SP, RM, and FA in a mass ratio of 10:3:18, a 2.0 mol/L NaOH solution is employed. The liquid-to-solid ratio is maintained at 20:31, and the sand-to-binder ratio is set at 3:1. The substitution of 12.28% SDW to NaOH solution improves the resistance to drying shrinkage and long-term mechanical strength development of the AACs. Interestingly, the addition of SDW does not affect the product compositions due to the generation and decomposition of organic acid salts from organic impurities in the acidic SDW during long-term curing at room temperature. These findings provide valuable insights for the sustainable recycling of bioresources and solid wastes containing silicon-aluminum in construction materials.

## Introduction

The intention of the Paris 2024 Olympics to construct the Olympic and Paralympic Villages using bio-based materials exemplifies the current trend towards environmentally friendly and sustainable building materials [1]. Unfortunately, the production of cement results in the release of significant amounts of carbon dioxide (CO_2_) gas, which is harmful to the environment [2, 3]. To put it into perspective, for every 600 kg of cement produced, approximately 400 kg of CO_2_ gas is emitted [4, 5]. In response to the urgent need to reduce emissions, the cement industry is required to achieve a 16% reduction by 2030. This emphasizes the importance of exploring alternative cementitious materials that are both environmentally friendly and sustainable, aligning with the climate change objectives outlined in the Paris Agreement. Alkali-Activated Material (AAM), which is a polymeric inorganic cementitious substance, has been identified as a feasible alternative to traditional cement [6]. AAMs, which possess a stable network structure created via alkali activation, are transforming from non-renewable resources to industrial solid residues such as slag, red mud, and fly ash [7, 8]. Solid waste comprises a wide range of materials, including agricultural, industrial, and household waste. However, industrial solid waste has a fluctuating chemical composition that requires precise balancing with other components to maintain the structural as well as mechanical stability of alkali-activated composites (AACs) [9]. Blending and implementing multi-solid wastes continues to be a substantial research obstacle, despite continuous attempts to enhance the properties of AACs.

Despite their potential as environmentally friendly alternatives, AACs face several disadvantages including high density, weight, and cost, as well as susceptibility to brittle fracture damage under strain [10]. However, a potential solution to this issue is the use of lightweight bio-composites, which are formed by incorporating plant aggregates into AACs [11]. This approach takes advantage of the natural compatibility between binders and plant fibers, resulting in final products that have improved thermal insulation and heat retention properties, thereby reducing energy wastage [2]. Previous research that incorporated various plant fibers into cement has shown progress in the development of eco-friendly construction materials [12–14].

Sawdust, a byproduct of agriculture and industry, is considered a suitable option for various applications [13]. However, it also poses environmental challenges due to its limited disposal capacity in landfills and significant greenhouse gas emissions when burned [15]. The United States and China, in particular, generate millions of tons of wood ash each year, which raises concerns about its proper recycling [16]. To address these issues, it is proposed that sawdust be utilized in cement-based composites as a sustainable method for effectively managing wood waste [17].

Extensive research has been conducted on the use of chemical treatments, particularly alkali treatment, to enhance the properties of natural fibers and debris before they are used in composites [18]. Alkali treatment has been found to effectively make natural fibers and debris hydrophobic, thereby improving the characteristics of composites [19, 20]. However, the environmental impact of the alkaline wastewater generated during these processes, which is often disposed of, continues to be an uninvestigated ecological issue.

To tackle the aforementioned problems, this study examined the viability of using alkali-treated sawdust particle (SDP) as aggregate additions for the manufacture of alkali-activated composites (AACs) by including various solid wastes such as fly ash, slag powder, and red mud. The physical and chemical characteristics of these composites were also evaluated. Then, this study examined the impact of sawdust wastewater (SDW) on the physical and mechanical characteristics of AACs. In addition, the microstructure and products were analyzed by a combination of SEM, EDS, XRD, and FTIR techniques to determine the mechanism by which SDW affects the system. This study investigated the application of SDP and SDW in solid waste-based AACs. The results will provide crucial knowledge about the recycling of bioresources, solid waste fuels, and the improvement of clean manufacturing methods.

## Materials and methods

### Raw materials

**Fig 1:**
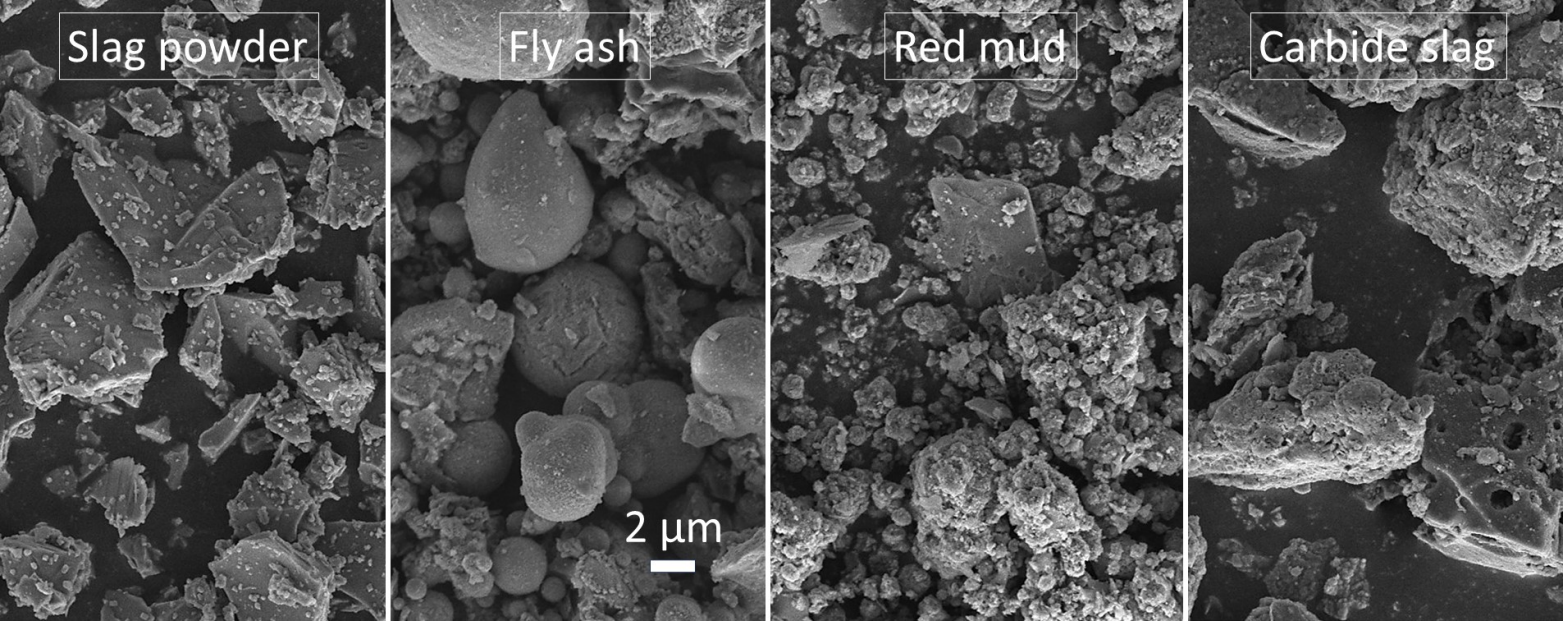
SEM images of binder materials with an identical magnification.

The manufacture of AACs included the inclusion of either sawdust or sawdust wastewater. The various materials include binder materials, alkali-activated solutions, alkali-treated solutions, sawdust particles (SDP), sawdust wastewater (SDW), and fine aggregates. Table 1 presents the chemical components of binder materials ascertained using X-ray fluorescence (XRF) analysis. Fig 1 depicts the structural characteristics of binder materials.

**Table 1:** Chemical components of binder materials: Slag powder (SP), red mud (RM), carbide slag (CS), and fly ash (FA). (Mass%, by XRF method).

Materials	SiO_2_	Al_2_O_3_	CaO	MgO	Fe_2_O_3_	K_2_O	SO_3_	Na_2_O	Others	Loss on Ignition (1000°C)
**SP**	35.1	16.2	33.6	11.1	—	—	—	—	4.1	—
**RM**	27.5	28.4	2.5	0.2	25.8	0.1	0.8	14.7	—	—
**FA**	46.1	23.2	5.4	2.6	8.0	1.8	0.8	—	4.4	7.6
**CS**	8.7	0.5	62.6	1.7	1.0	—	—	—	—	25.4

#### Binder materials

Four primary binder materials were employed: slag powder (SP), red mud (RM), fly ash (FA), and carbide slag (CS). RM and FA were introduced into alkali-activated SP-based materials to ameliorate cracking during curing, crucial for enhancing AAC durability [21]. Additionally, CS was utilized to mitigate drying shrinkage and maintain stable mechanical strength development in alkali-activated SP-based materials [22]. Consequently, this study amalgamated multi-solid wastes as two system binders. The nature of the four types of binder materials is shown as follows:

Slag Powder (SP): The SP of grade #S95 has a fineness of 800 mesh and a specific gravity of 2.67. It has a pH value of 11.45 and an electrical conductivity (EC) of 0.44 mS/cm when measured at a solid-water mass ratio of 1:5. The SP originated in Gongyi City, located in Henan Province, China.

Red Mud (RM): RM demonstrated an uneven granular structure, fine particles, a loose structure, and high alkalinity at a fineness of 500 mesh. The key metrics consisted of a specific gravity of 2.56, pH level of 10.52, and EC of 2.18 mS/cm. The primary chemical constituents were SiO_2_, Al_2_O_3_, and Fe_2_O_3_. The origin was Gongyi City, China.

Fly Ash (FA): This material satisfied Class II fly ash requirements and had a fineness that was in line with the low-calcium fly ash classification. The specific gravity was 2.25, while the pH and EC were measured to be 8.30 and 2.75 mS/cm, respectively. The FA was obtained from a power plant located in Handan City, Hebei Province, China.

Carbide Slag (CS): CS is a residual material generated during the manufacturing of acetylene using calcium carbide [7]. It consists of around 62.6% calcium oxide (CaO). Obtained from a chemical facility in Qinghai Province, China, this substance was produced by the wet method of creating acetylene gas. CS has a specific gravity of 1.80, a specific surface area of 420 m^2^/kg, 40% of it passes through a #325 sieve, and its pH value is 10.55 with 100% water content. Fig 1 illustrates that SP has irregularly angular granules, whereas RM exhibits a combination of small particles and flakes. On the contrary, FA displays spherical particles and irregular constituents, whereas CS showcases particles with an irregular porous structure.

#### Alkali-activated solutions

Two alkaline activators were prepared, consisting of 2.0 mol/L Na_2_SiO_3_ and 2.0 mol/L NaOH solutions. These concentrations, known to effectively activate alumina-silicate materials and synthesize cementitious compounds, were selected [18]. The Na_2_SiO_3_ pellets, an alkaline activator with analytical reagent quality, were dissolved in tap water to create the 2.0 mol/L Na_2_SiO_3_ solution [23]. Simultaneously, a 2.0 mol/L NaOH solution was generated by dissolving a NaOH pellet in tap water. Both Na_2_SiO_3_ and NaOH pellets, procured from Tianjin Aopusheng Chemical Co., Ltd. (China), possessed an analytical purity exceeding 98%. Additionally, the tap water (pH = 7.05 ± 0.05) originated from Handan City, Hebei Province, China. These alkaline solutions were allowed to reach room temperature before sample preparation to mitigate the influence of elevated temperatures.

#### Fine aggregate

The river sand, which acts as the fine aggregate, has a fineness modulus of 2.62 and a bulk density of 2.64 g/cm^3^.

#### Alkali-treated solution

To examine the impact of treatment duration, a 2.5 mol/L concentration of NaOH solution treated the sawdust particles [1]. This high concentration was chosen, following conventional alkaline solutions of 1%–15%, to expedite the decomposition and leaching of organic substances. The 2.5 mol/L NaOH treated solution was prepared using analytically pure NaOH powders (purity > 98%) with tap water, mirroring the preparation of alkali-activated solutions. Furthermore, the tap water (pH = 7.05 ± 0.05) was sourced from Handan City, Hebei Province, China.

#### Sawdust particles (SDPs) and sawdust wastewater (SDW)

Poplar sawdust particles, derived from a local timber industry, possess a bulk density of 0.136 g/cm^3^ and an absorption coefficient of 245%. With lengths ranging from 1.0–3.0 mm, these SDPs were dried in an oven at 105°C for over four hours to eliminate moisture [24]. Chemical and physical characteristics varied based on the plant species, with poplar SDPs considered solid wastes. These dried SDPs underwent soaking in a 2.5 mol/L NaOH solution for durations of 0.5, 2.0, 6.0, and 12.0 h, respectively. Following this treatment, the SDPs were dried in an oven for six hours without being washed, aiming to minimize wastewater production. In contrast, untreated dried SDPs served as the control group.

**Fig 2:**
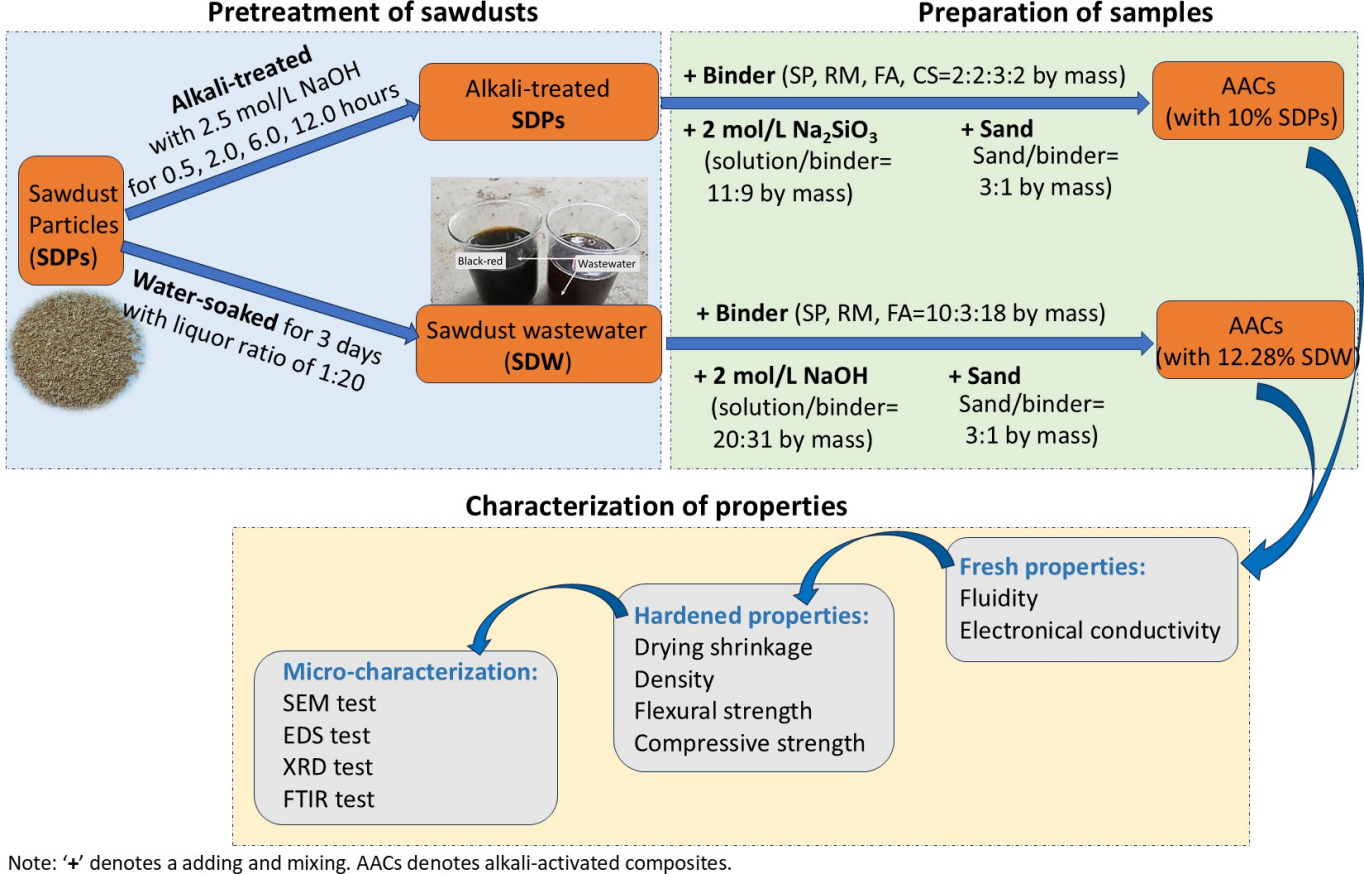
Schematic diagram of the flow work.

SDPs were immersed in tap water (liquor ratio of 1:20 by mass) in glass containers for three days. After filtration, the resulting SDW had a pH of 6.70 and an EC of 286 μS/cm. The color change from colorless to black-red in SDW indicated the leaching of organic impurities (Fig 2).

### Sample preparation

The investigation aimed to explore the impacts of three primary factors. Using a Na_2_SiO_3_ solution, the effects of SDP content and alkali-treated time were examined in the SP-RM-FA-CS tetradraplex system, as shown in Fig 2. Additionally, the influence of SDW was explored in the SP-RM-FA ternary system activated by a NaOH solution. Employing two alkali-activated systems enhances the experimental evidence for the application of SDP and SDW in inorganic cementitious composites with multi-solid wastes. The specific sample proportions are detailed in Table 2, and samples without river sand were prepared for corresponding test determinations. Specifically, the ternary system was compared to the tetradraplex system, with the removal of CS from the binder and substitution of the Na_2_SiO_3_ solution with a NaOH solution. CS removal simplifies gel product determination due to complex chemical compositions from the multi-solid waste mixture. The shift to a NaOH solution for alkali activation serves to reduce the influence of excess SiO_3_^2-^ ions and maintain consistency with the alkali-treated solution, facilitating the relationship determination between the alkali-activated and alkali-treated solutions.

**Table 2:** Designed mixing proportions of sawdust particles (SDPs) and sawdust wastewater (SDW) applied in alkali-activated solid waste-based materials. Na_2_SiO_3_ solution is 2.0 mol/L and NaOH solution is 2.0 mol/L as alkaline activator.

Sample number	Binder (SP: RM: FA: CS) by mass	Sand /Binder ratio	Na_2_SiO_3_ solution /Binder ratio	SDPs /Binder ratio	(SDPs) Treated time (hour)
**MS1**	2:2:3:2	3:1	13:9	0.0%	0.0
**MS2**	2:2:3:2	3:1	13:9	3.3%	0.0
**MS3**	2:2:3:2	3:1	13:9	6.7%	0.0
**MS4**	2:2:3:2	3:1	13:9	10.0%	0.0
**MS5**	2:2:3:2	3:1	13:9	13.3%	0.0
**MS11**	2:2:3:2	3:1	11:9	10.0%	0.0
**MS12**	2:2:3:2	3:1	11:9	10.0%	0.5
**MS13**	2:2:3:2	3:1	11:9	10.0%	2.0
**MS14**	2:2:3:2	3:1	11:9	10.0%	6.0
**MS15**	2:2:3:2	3:1	11:9	10.0%	12.0
**Sample** **number**	**Binder** **(SP: RM: FA) by mass**	**Sand** **/Binder** **ratio**	**NaOH solution** **/Binder ratio**	**SDW substitution** **(SDW/NaOH solution ratio)**	
**MW1**	10:3:18	3:1	20:31	0.00%	
**MW2**	10:3:18	3:1	20:31	6.14%	
**MW3**	10:3:18	3:1	20:31	12.28%	
**DW1**	10:3:18	2:1	18.8:31	0.00%	
**DW2**	10:3:18	2:1	18.8:31	6.14%	
**DW3**	10:3:18	2:1	18.8:31	12.28%	
**JW1**	10:3:18	—	16.4:31	0.00%	
**JW2**	10:3:18	—	16.4:31	6.14%	
**JW3**	10:3:18	—	16.4:31	12.28%	

The sample preparation was carried out based on the following process (Fig 2). Section one investigates the SDP dosage issue for the "MS*" series samples; Section two studies the alkali treatment duration issue of SDPs with the "MS1*" series samples. Section three introduces SDW into the composite materials using the "MW*" series samples. The development of the manuscript proceeds as follows: Section one first determines the appropriate amount of SDP addition. Then, Section two treats the SDPs with alkali. Furthermore, Section three uses SDW to synthesize composite materials. Finally, in Section four, micro-experiments are further conducted on samples prepared using SDW, combined with the control group (samples without SDW addition), to analyze the effect mechanism of the SDW (Fig 2). The preparation methods of samples were shown as follows:

#### Tetradraplex system

Various binder materials were initially blended thoroughly, followed by the gradual addition of a Na_2_SiO_3_ solution with slow-speed stirring for one minute. After introducing river sand, the stirring process continued at a slow speed for an additional minute. Subsequently, different SDP contents (Table 2) were added, and the mixture underwent slow stirring for one minute, followed by a fast-stirring process for one minute, resulting in fresh mortar. After assessing the fresh properties, the mixture was poured into a steel mold in two layers. Vibrations were applied for sample consolidation and bubble removal, and the samples were then open-air cured at room temperature (23±2°C) and humidity (>50%) for one day. The binder mixing method in the Tetradraplex System is referenced from previous research [18].

#### Ternary system

Three types of binder materials were meticulously mixed, and NaOH solution was added in one go. Stirring occurred at a slow speed for two minutes, and then river sand was introduced, with continued slow-speed stirring for one minute. Subsequently, various substitution ratios of SDW were considered (Table 2), and an additional NaOH solution was introduced to maintain a constant water-to-solid ratio. After a slow-speed stirring process of two minutes, fresh mortars were obtained. The fresh mortars were poured into steel molds after vibration and subjected to open-air curing at room temperature (23±2°C) and humidity (>50%) for one day. The binder mixing method in the Ternary System is referenced from previous research [25].

After that, steel molds were then removed, and samples were further maintained with identical curing until reaching the required testing age for the two systems.

In the designed mixing ratio (Table 2), a 0–13.3% SDP/Binder ratio was selected based on previous studies. Prior researchers [26–28] identified a 10% replacement of SDP as the ideal amount for producing sawdust concrete.

### Experimental methods

To analyze workability and chemical reaction potential, fresh sample properties such as fluidity and electrical conductivity (EC) were measured. The investigation extended to understand the changing law of hardened properties concerning SDP content, treated time of SDPs, and SDW contents by examining drying shrinkage, density, and mechanical strength (flexural and compressive strengths). Additionally, SEM-EDS, XRD, and FTIR tests were conducted to explore the influence of SDW on the properties, products, and microstructures of AACs through a synthetic mechanism of hardened samples. Herein, all mortar samples were tested for electrical conductivity and fluidity. Mortar samples from the "MS*" series, as well as MW1, MW2, and MW3, were tested for density and mechanical strength. Mortar samples from the "MS*" series, as well as DW1, DW2, and DW3, were tested for drying shrinkage. Microscopic tests were conducted using JW1 and JW3.

The experiments were conducted based on the laboratory of Hebei University of Engineering (Handan, China) and the Shiyanjia testing platform of Hangzhou Yanqu Information Technology Co., Ltd. (Hangzhou, China). Hebei University of Engineering is responsible for the fresh and hardened performance tests of the materials (Website: https://fxzx.hebeu.edu.cn/index.htm). Hangzhou Yanqu Information Technology Co., Ltd. is a third-party testing company and is responsible for the micro-detection of the materials through cooperative or employment relationships (Website: https://www.shiyanjia.com/).

#### Determination of fresh and hardened properties

*(1) Electrical conductivity and fluidity*. Electrical conductivity (EC) was measured by using an electrical conductivity meter (DDS-307A) that measures up to 100 mS/cm with a resolution of 0.01 mS/cm. The measurements were taken at six different locations within the mixing pot using a reliable 10 cm^-1^ electrode probe to ensure a representative sample. The electrodes were inserted at depths of 1 to 2 cm, and the average EC values were calculated based on the collected data.

Afterward, the fresh mixture was carefully placed in a truncated conical container. The flow diameter of the mortar was determined using the jump table methodology specified by GB/T 2419–2005 [29]. The average flow diameter was calculated based on the analysis of three parallel samples. These measurements were taken after the mixing process was completed and before the mixture was poured into the mold, ensuring the accuracy and reliability of the results.

*(2) Densities and mechanical strengths*. The density measurements were performed at distinct intervals of 6 hours, 28 days, 60 days, and 90 days, employing the highly regarded mass-to-volume method [18]. To guarantee precise and consistent outcomes, the density values were obtained from six sets of parallel specimens.

To discern the age-dependent effects and stability of the alkali-activated samples, the flexural and compressive strengths were evaluated. To conduct these assessments, samples featuring dimensions of 40 mm × 40 mm × 160 mm were subjected to rigorous testing at three distinct time points: 28, 60, and 90 days. The flexural strength was determined based on the average of three parallel results, while the compressive strength was calculated from the average of six parallel results. A servo-control testing machine (DYE–300–10, Tongli, Hebei, China) boasting a maximum loading capacity of 300 kN was employed. The compression strength test was conducted under a controlled loading rate of 2400 N/s, while the flexural strength test was executed at a loading rate of 50 N/s, following established protocols elucidated in the esteemed literature [22, 30].

*(3) Drying shrinkage*. To gauge the drying shrinkage phenomenon, samples measuring 25 mm × 25 mm × 280 mm were monitored till 90 days, employing a high-precision ratio-length meter (BC156-300, Lisheng, China) that adhered to the exacting standards outlined in the revered Chinese standard JC/T 603–2004 [31]. To ensure unwavering precision and reliability, an assessment was conducted on six parallel samples repeatedly under identical conditions.

#### Determination of micro-characteristics

*(1) Morphology and microstructure*. The morphology and microstructure of the 90-day samples and binder materials were analyzed by using scanning electron microscopy (SEM) with energy-dispersive spectroscopy (EDS). The SEM instrument used was TESCAN MIRA LMS from the Czech Republic, and the EDS instrument used was Smartedx from Oxford, England. To improve surface conductivity, a layer of gold was applied to the specimens. The EDS spectra analysis was then performed to examine and analyze the elemental composition in specific regions of the 90-day samples.

*(2) Mineral phases and products*. Mineral compositions in the samples were identified by using X-ray diffraction spectroscopy with Cu Kα radiation. The scanning rate was set at 2°/min, covering the range of 10° to 80° 2θ. After drying the specimens in an oven for 48 hours at 45°C, they were ground to achieve a particle size of 2.0 μm. Testing specimens were prepared by mixing 1.3 ± 0.001 mg of the 90-day samples and binder materials with 130 mg of KBr reagent, followed by applying a pressure of 20 MPa. Fourier Transformation Infrared Spectroscopy (FTIR) was then conducted by using a Nexus 8 instrument. The FTIR analysis covered the range of 400 to 4000 cm⁻^1^, and it involved analyzing characteristic vibration peaks and product analysis to gain valuable insights [32].

## Results and discussion

### Influence of sawdust content on the properties

#### Fluidity and electrical conductivity

**Fig 3:**
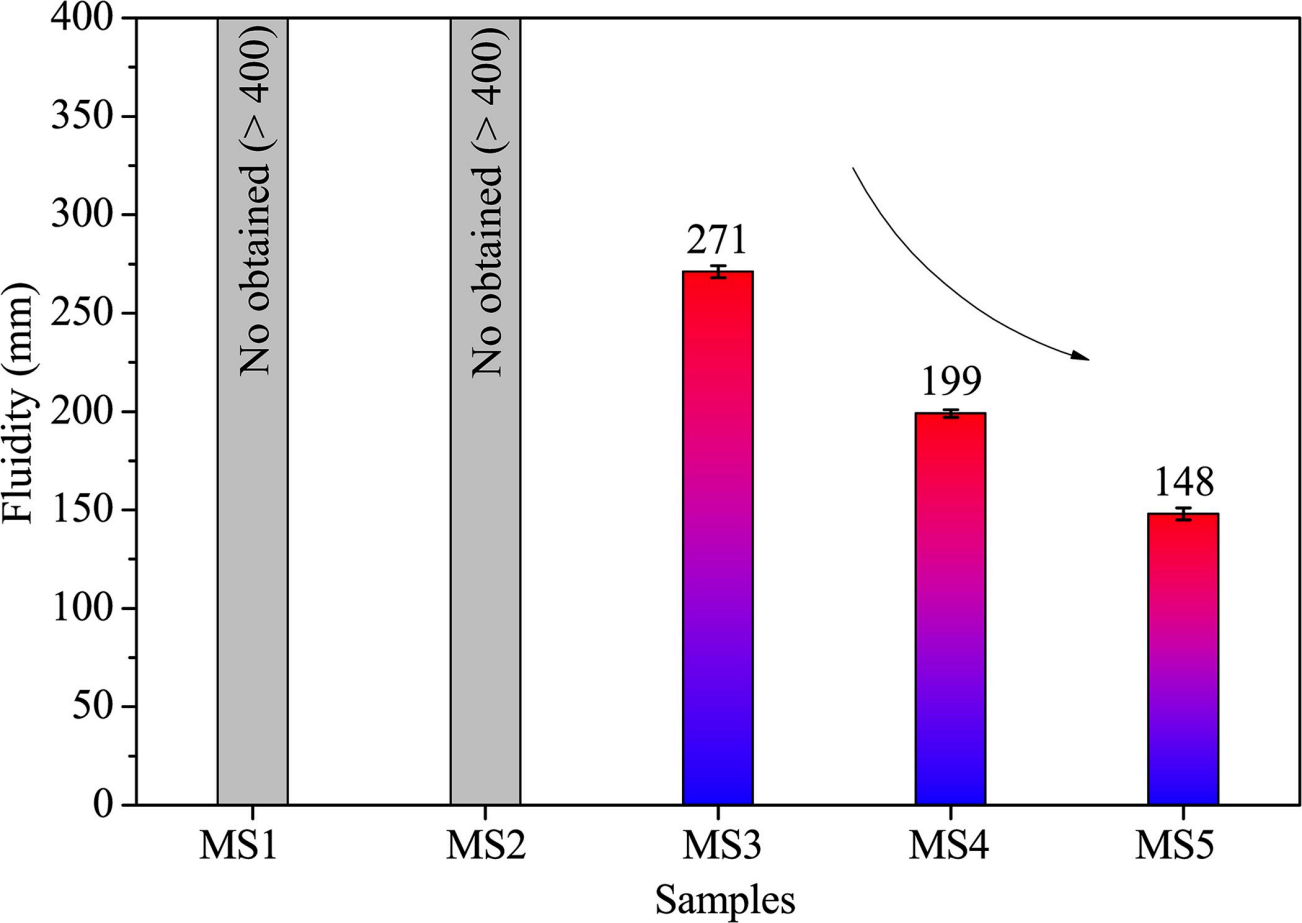
Fluidity of fresh samples with varying SDP contents.

In Fig 3, fluidity results reveal a substantial decrease as SDP content rises from 0% to 13.3%. Notably, samples MS1 and MS2 exhibited excessive fluidity, causing data unattainability as the fresh mortar flowed off the table during vibration. However, data for samples MS3, MS4, and MS5 were successfully obtained. Sample MS5 exhibited a 45.4% decrease in fluidity compared to MS3, attributed to the hygroscopic nature of SDPs, a wood-derived material with rapid water absorption (245%). This decline aligns with similar reductions in fluidity observed with increasing bamboo sawdust content [1].

**Fig 4:**
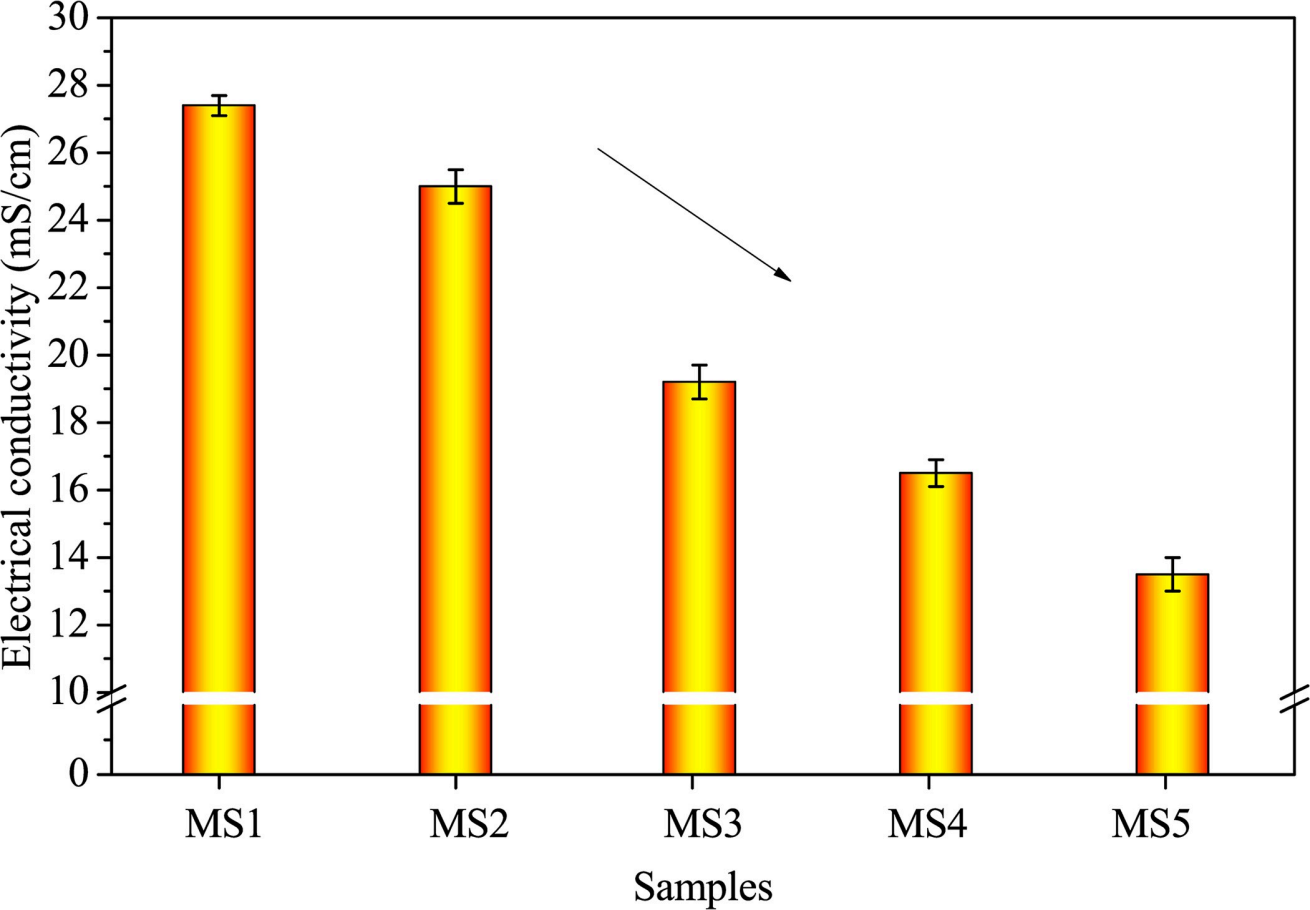
Electrical conductivity of fresh samples with varying SDP contents.

Fig 4 illustrates the electrical conductivity (EC) variations. An increase in SDP incorporation correlates with decreased EC. This can be attributed to several factors. First, the replacement of more gelling material by SDPs results in fewer reactive substances. Second, SDPs, being a wood-derived waste, contain organic substances that dissolve in water or an alkaline environment, reducing conductivity due to molecular form [18, 33]. Third, the alkaline environment diminishes the contact and reaction rates of atomic groups containing silicon and aluminum, as well as free ions such as Na^+^ and OH^-^, leading to a lower alkali-activated reaction rate.

#### Drying shrinkage and density

**Fig 5:**
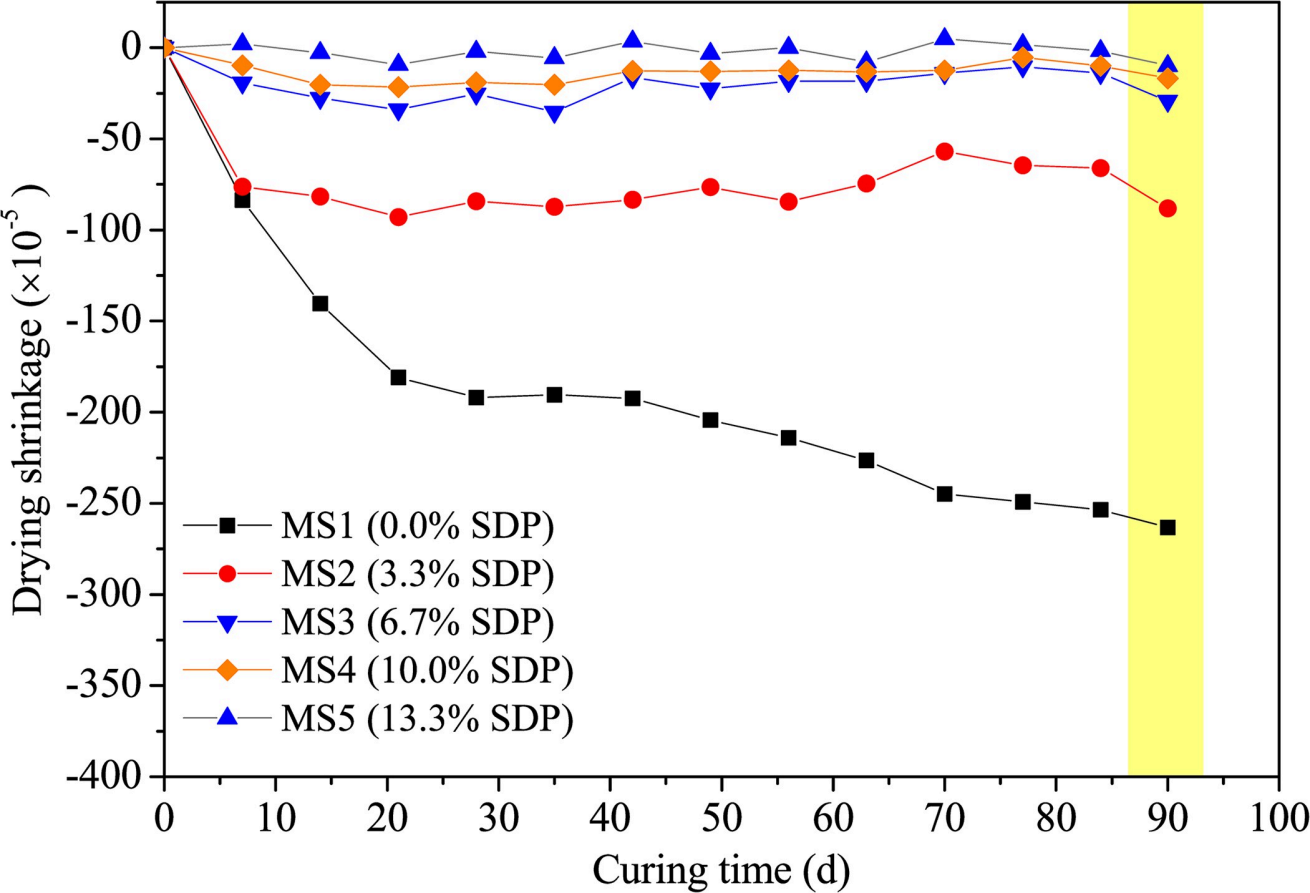
Drying shrinkage vs. curing times.

**Fig 6:**
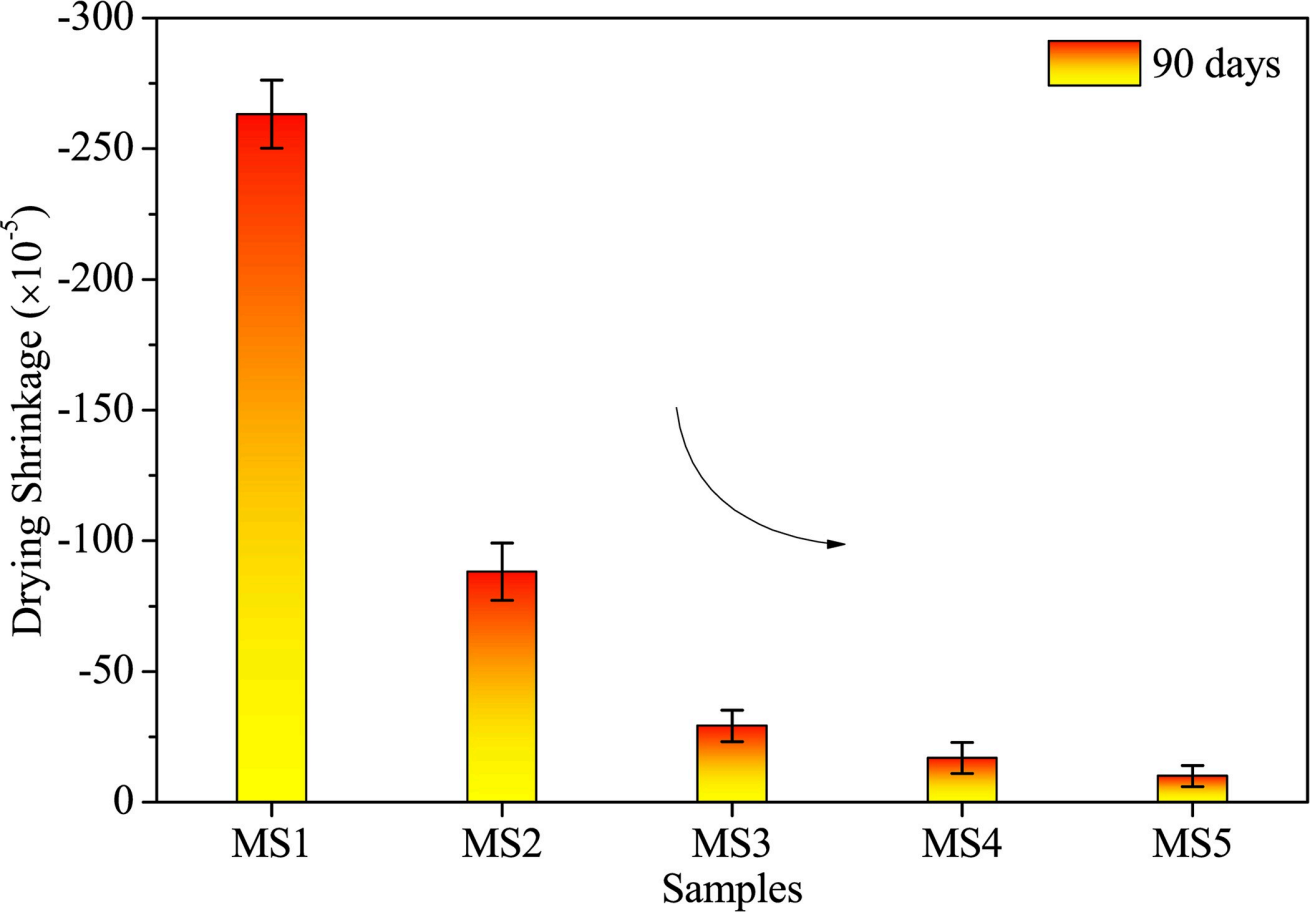
Drying shrinkage vs. sawdust particle contents.

Fig 5 illustrates the drying shrinkage and density variations in samples with different SDP contents. In Figs 5 and 6, a substantial reduction in 90-day drying shrinkage is evident in alkali-activated samples with SDPs at 3.3%, 6.7%, 10.0%, and 13.3% by mass compared to the SDP-free control. The sample with 3.3% SDPs exhibits a 66.5% reduction in drying shrinkage compared to the control, while the 13.3% SDPs sample shows an 88.7% decrease compared to the 3.3% SDPs sample. This reduction is attributed to the water absorption and expansion characteristics of SDPs, functioning as a filler and mitigating shrinkage during alkali activation.

**Fig 7:**
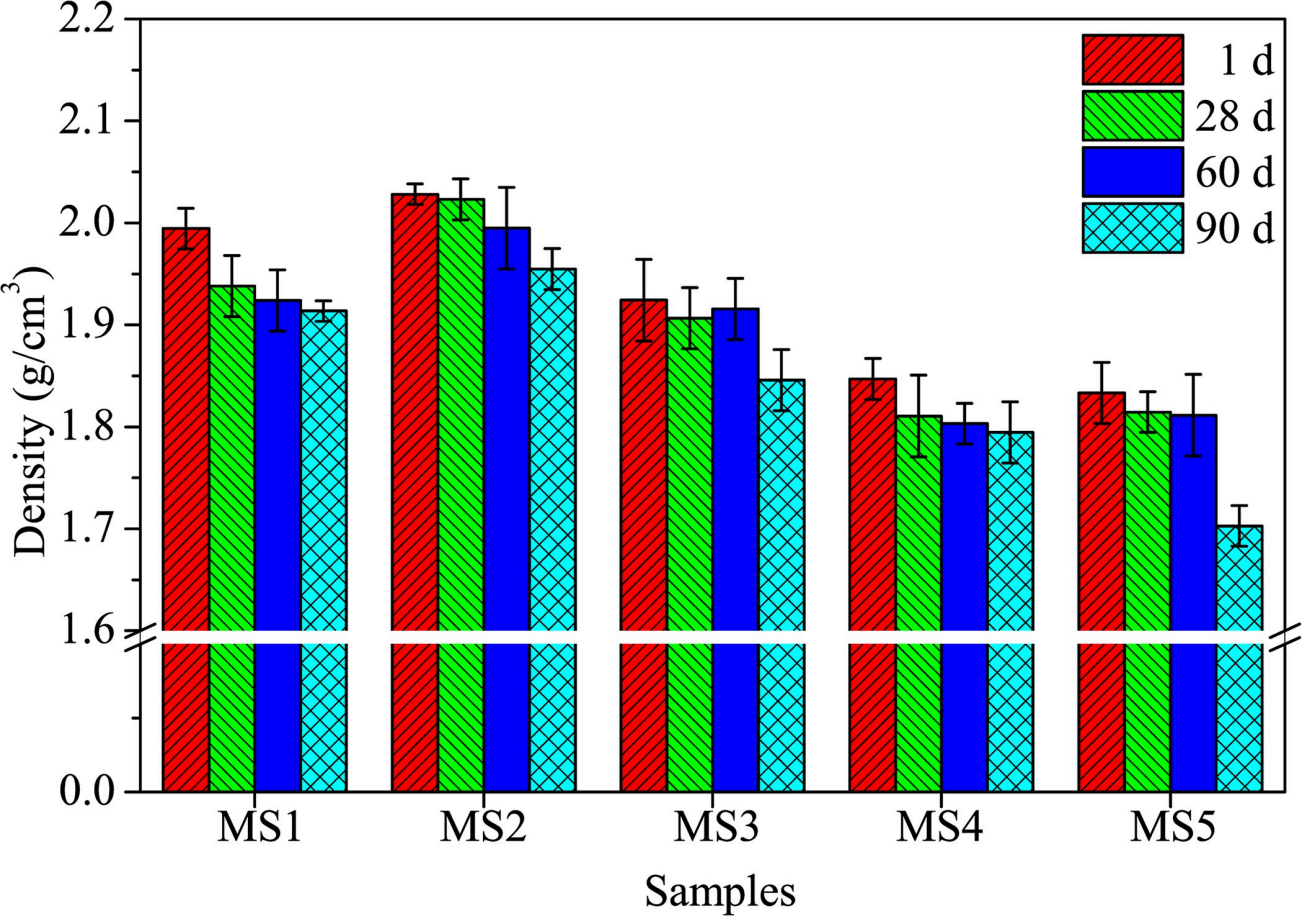
Density vs. SDP contents.

Furthermore, the density of alkali-activated samples decreases with curing age due to moisture loss (Fig 7) [18, 34]. The density also decreases with increasing SDP content because SDPs have a lower specific gravity, which acts as a substitution effect. The sample with 10.0% SDPs has a density of 1.79 g/cm^3^ at 90 days, which is higher than the density of alkali-activated sample with plant wastewater (1.15 g/cm^3^) [18] and sisal fiber-reinforced composites (1.45 g/cm^3^) [35]. However, it is lower than the density of NaOH-activated materials (CON) utilizing SP, FA, RM, and steel slag (2.08 g/cm^3^) [18], and the referenced density range of alkali-activated mortar with FA, soda residue, and CS (density range of 2.00–2.30 g/cm^3^) [22, 36]. The reduction in density may be due to increased porosity from entrapped air and the lower density of natural SDPs compared to the alkali-activated solid waste-based matrix, as reported by Ye et al. [37, 38].

#### Mechanical strengths and morphology

**Fig 8:**
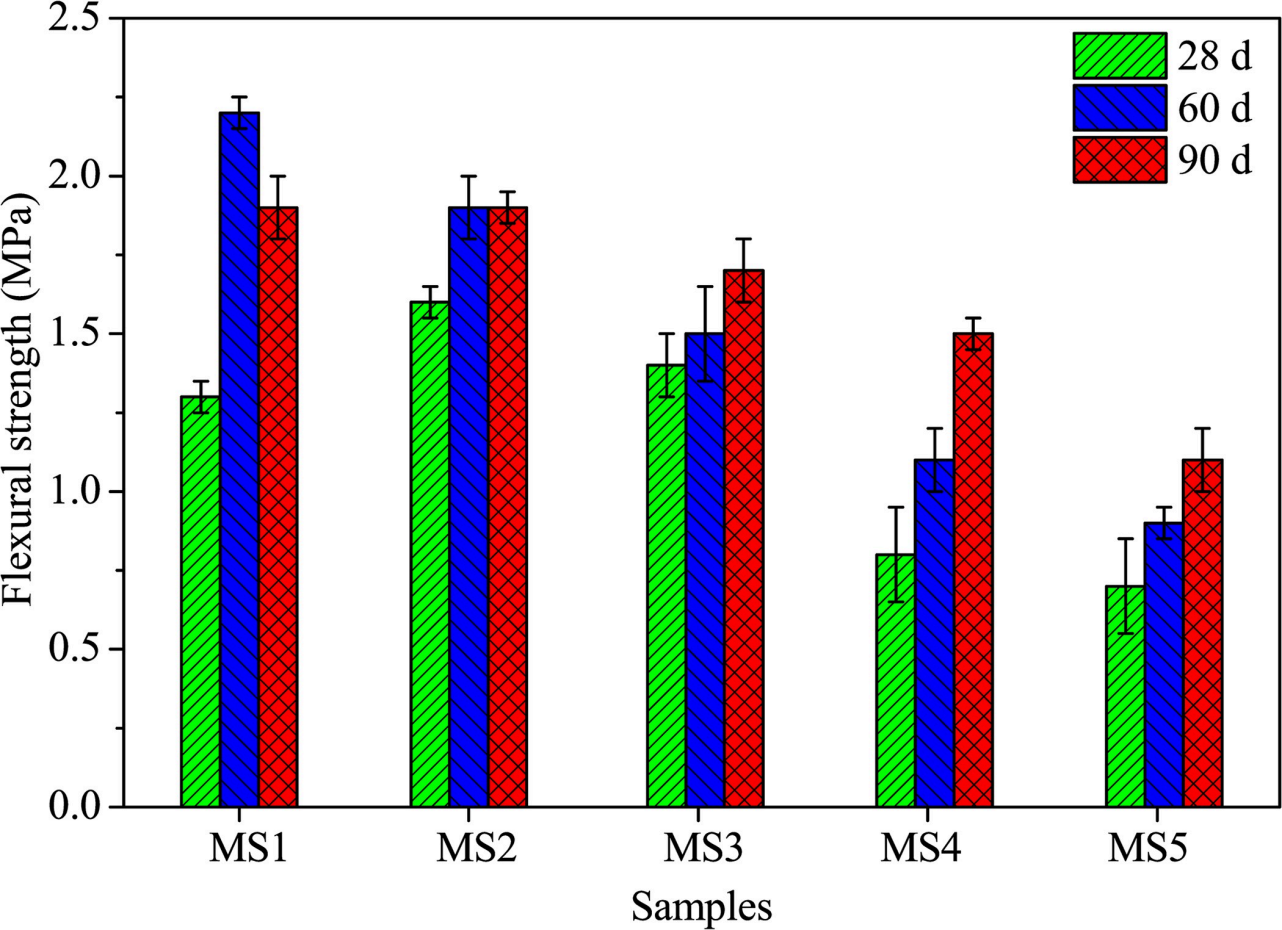
Flexural strengths of alkali-activated composites cured for 28, 60, and 90 days.

The variation of flexural and compressive strengths with curing age for AACs under different SDP contents is depicted in Fig 8. A progressive increase followed by a decline in flexural strength is observed at 28 days with rising SDP content, peaking at 1.6 MPa for a 3.3% SDP level (Fig 8). However, at 60 and 90 days, the flexural strength diminishes with increasing SDP content. Particularly, at a 13.3% SDP level, the flexural strength decreases by 59.1% at 60 days and 42.1% at 90 days compared to the control. Unlike the control, the inclusion of SDPs mitigates the strength regression phenomenon. This regression, attributed to gaps from raw material hydration or internal retention due to shrinkage [39], is alleviated by reduced chemical bindings, decreasing cracks and gradually improving AAC strength with curing age.

**Fig 9:**
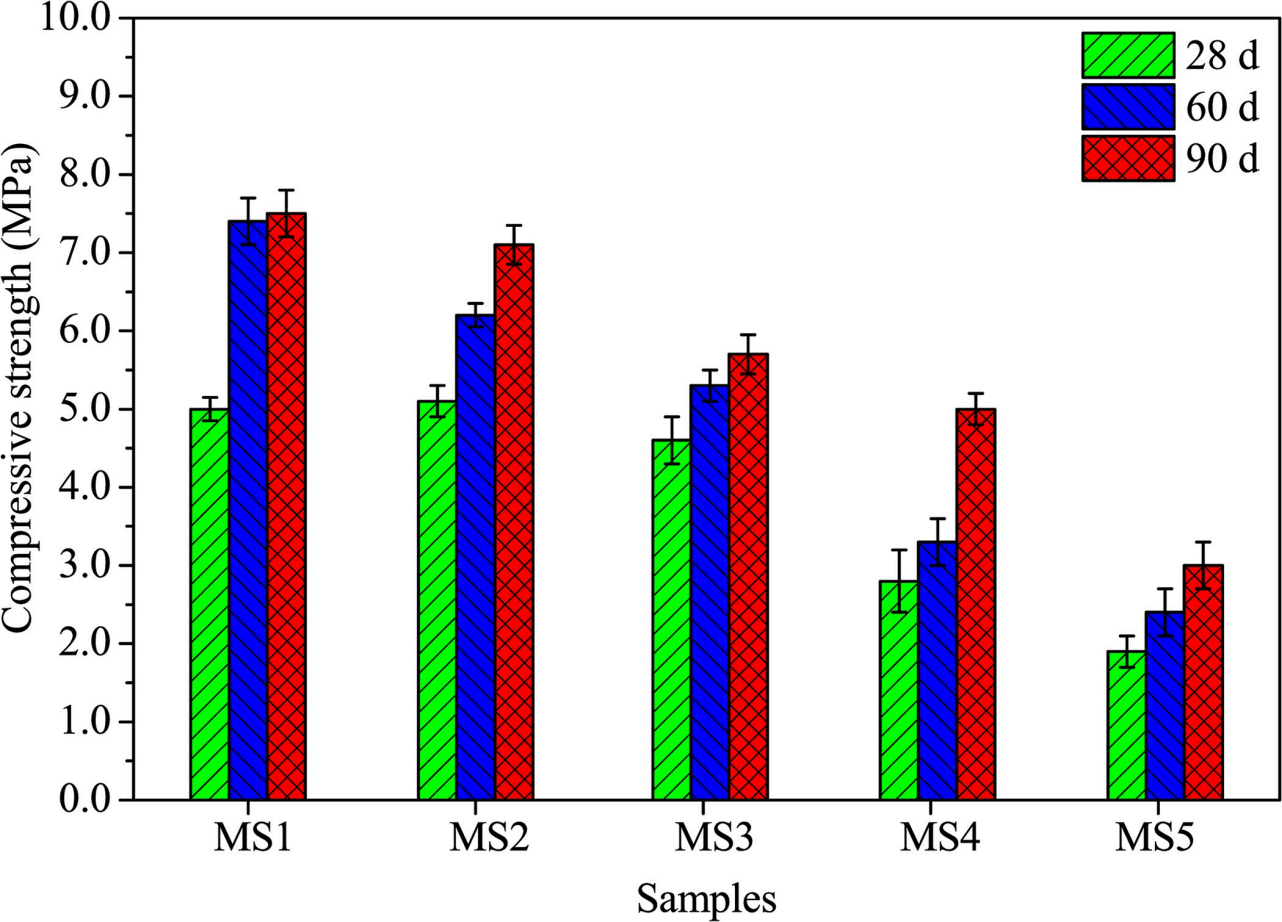
Compressive strengths of alkali-activated composites cured for 28, 60, and 90 days.

Similarly, Fig 9 illustrates a consistent trend in compressive strength with SDP content as observed in Fig 8. The maximum compressive strength of 5.1 MPa is attained at a 3.3% SDP level at 28 days. At a 13.3% SDP level, compressive strengths at 28, 60, and 90 days decrease by 62.0%, 67.6%, and 60.0%, respectively, compared to the control. The decline in strengths is attributed to the incorporation of low-strength SDPs into the matrix and the dissolution of non-cellulosic components, such as hemicellulose, pectin, polysaccharides, and lignin from SDPs in an alkaline environment, resulting in reduced structural bonding and increased porosity [18, 40].

Studies by Suliman et al. [26], Olutoge [41], Sasah, and Kankam [42] have confirmed that the flexural strength of cement-based blocks decreases as the sawdust content increases. Suliman et al. [26] achieved a compressive strength of 4.5 MPa by adding 15.0% SDP to the blocks. In this particular study, a 90-day compressive strength of 5.0 MPa was obtained with a 10% SDP content, which meets the Nigerian technical requirements of 3.5–10.0 MPa [2] and exceeds the minimal strength of 2.8 MPa in BS 6073 [43]. Therefore, the mechanical strength achieved in this study supports the potential of using sawdust-based composites, known as SDPs, as a replacement for sand in building material preparation, such as blocks and bricks made from alkali-activated solid waste.

**Fig 10:**
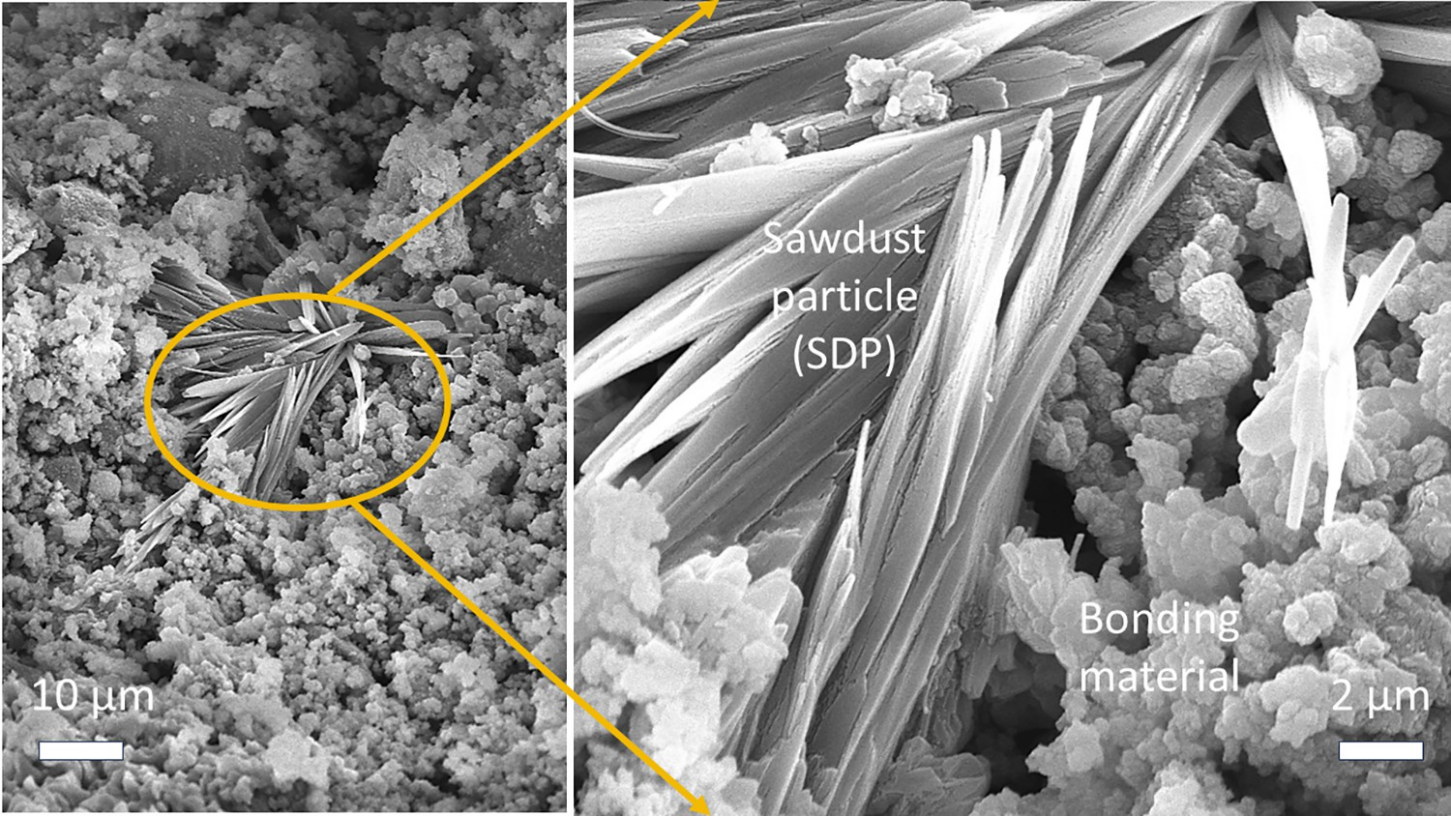
The SEM images of sawdust particles (SDPs) in the alkali-activated sample MS4 with 10.0% of SDP contents.

Fig 10 illustrates the morphology of AACs with a 10.0% SDP content. The insertion of SDPs into the AAC matrix is evident; however, the SDPs surface is smooth, establishing minimal bonding with the surrounding matrix. This observation indicates the ineffectiveness of the 2.0 mol/L Na_2_SiO_3_ solution in etching the SDPs surface during sample preparation. Consequently, the presence of SDPs adversely affects the mechanical properties, water absorption, and water resistance of AACs (as shown in S1 Fig). The increased SDP content not only reduces free water but also diminishes the reaction area between gelling particles [11]. This reduction results in a looser microstructure, lowering the relative content of product gels and the mechanical strengths of AACs incorporating SDPs as a filler.

### Influence of alkali-treated sawdust particles on the properties

The inclusion of SDPs is found to decrease fluidity, EC, density, drying shrinkage, flexural strength, and compressive strength in AACs. Further investigation is warranted to explore the alkali treatment of SDPs and their duration’s influence on AAC properties.

**Fig 11:**
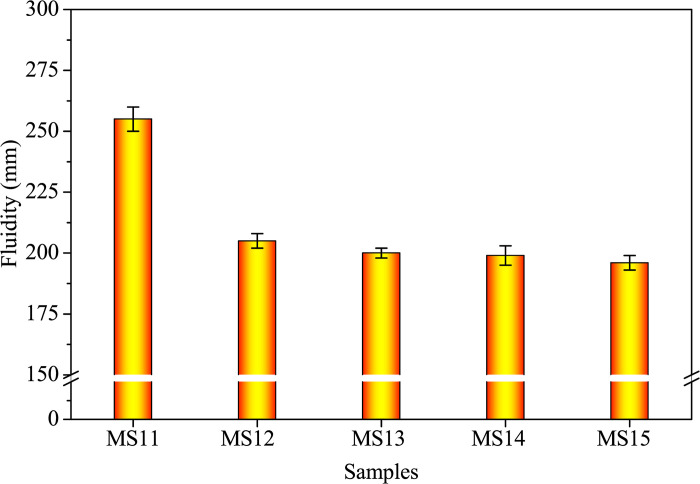
Fluidity of fresh samples with different alkali-treated durations of SDPs.

**Fig 12:**
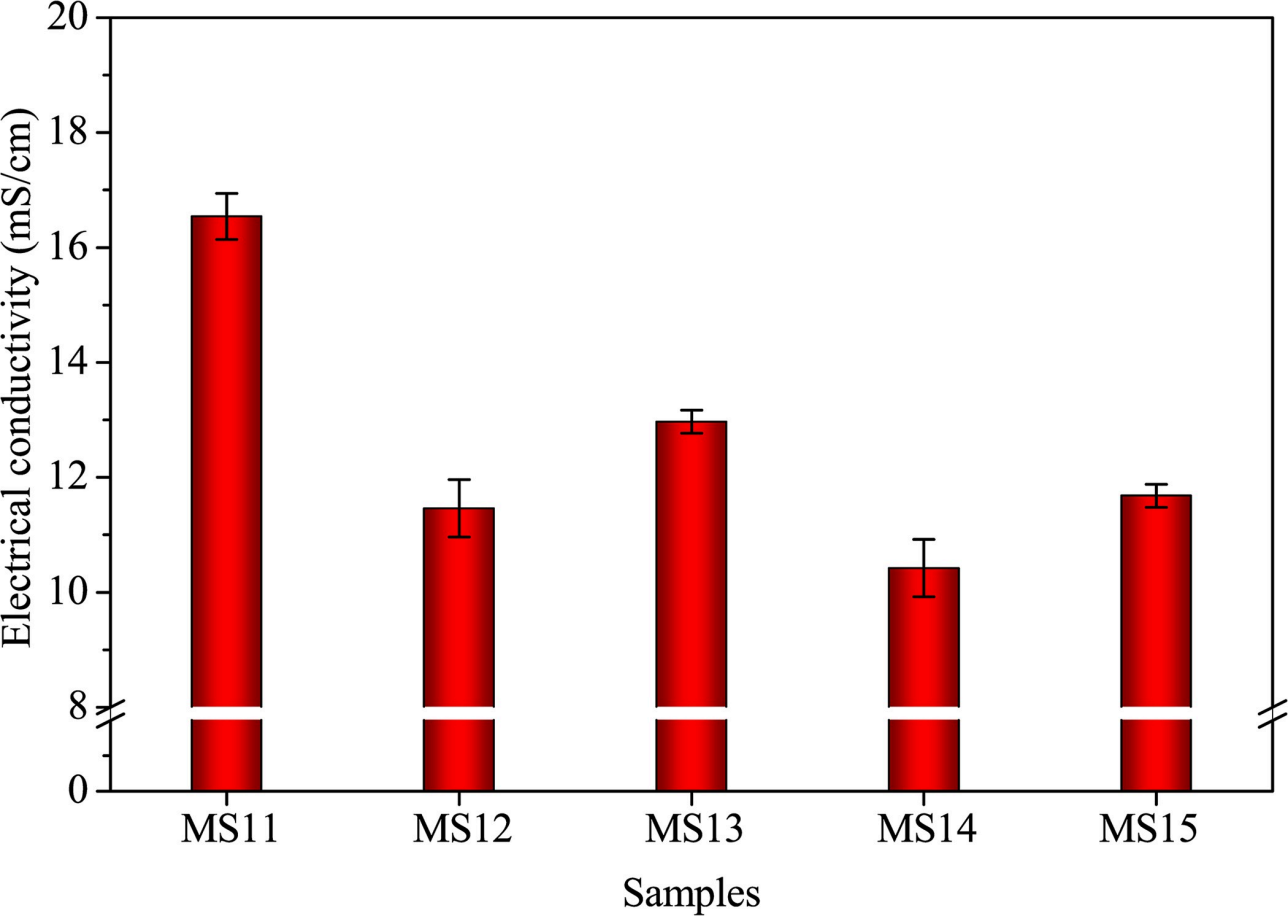
Electrical conductivity of fresh samples with different alkali-treated durations of SDPs.

Fig 11 illustrates the influence of varying alkali-treated durations (0.5, 2.0, 6.0, and 12.0 hours) on fluidity and EC at a 10.0% SDP level. The results demonstrate that a longer alkali-treated duration leads to decreased fluidity (Fig 11) and EC (Fig 12), highlighting the significance of surface bonding and the presence of organic impurities. The data suggests that a 0.5-hour treatment duration effectively removes the dissolved organic impurities from SDPs.

**Fig 13:**
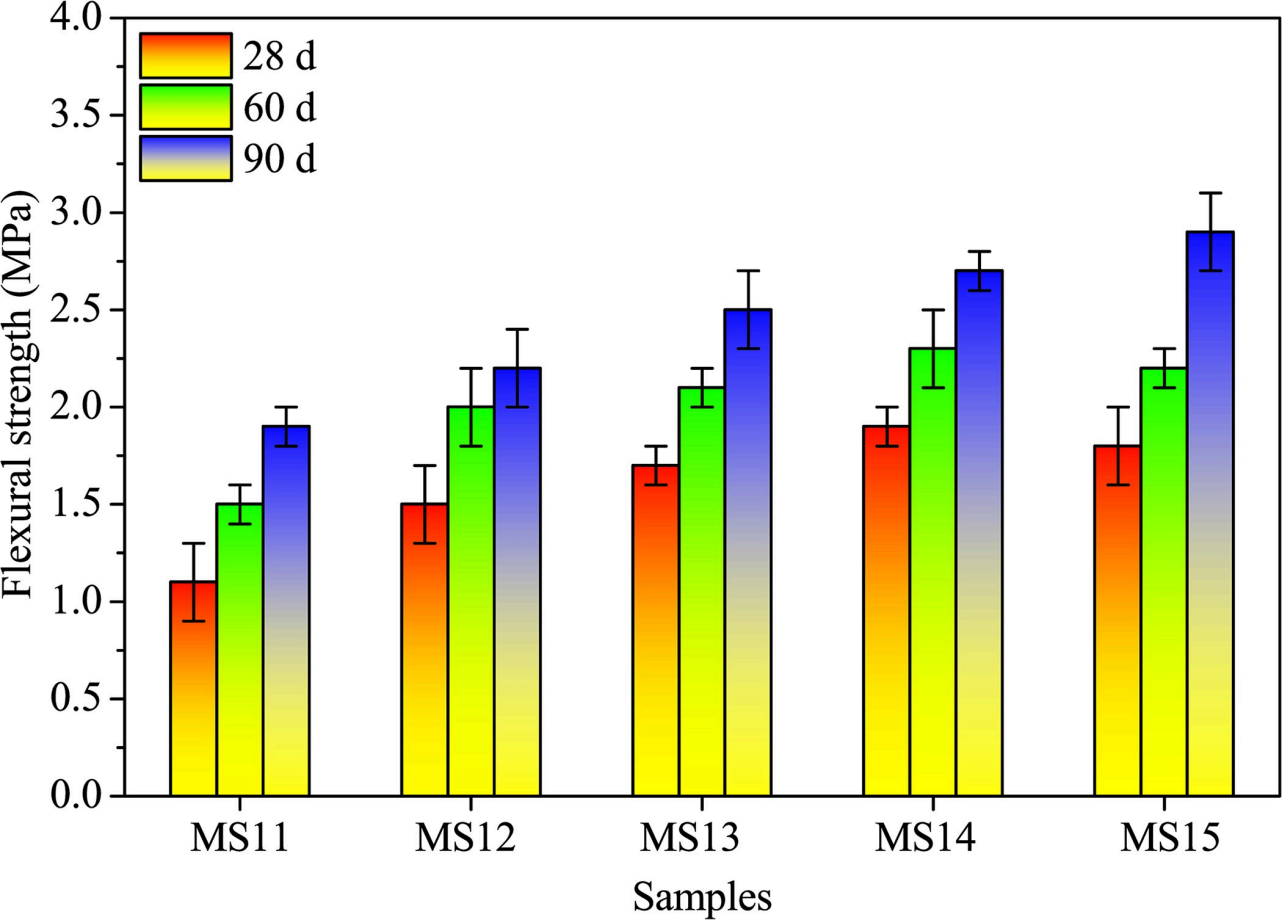
Flexural strengths of alkali-activated composites with different alkali-treated durations of SDPs when cured for 28, 60, and 90 days.

**Fig 14:**
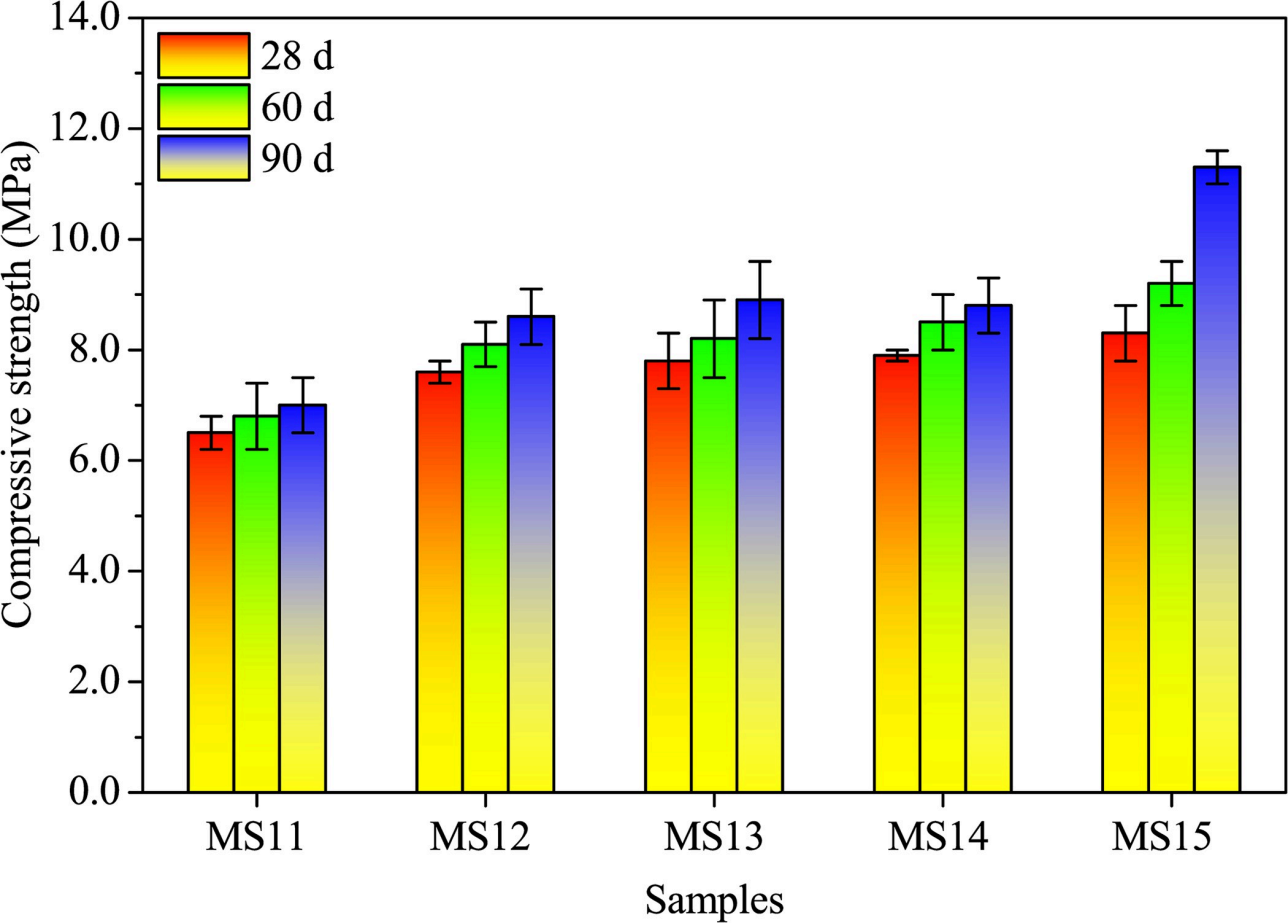
Compressive strengths of alkali-activated composites with different alkali-treated durations of SDPs when cured for 28, 60, and 90 days.

Figs 13 and 14 presents the flexural and compressive strengths at a 10.0% SDP level. Both strengths increase with curing age, showcasing the age-dependent effect of AAMs [8, 21]. Additionally, alkali treatment durations of 0.5, 2.0, 6.0, and 12.0 hours enhance these strengths, with a notable improvement observed as the alkali-treated duration extends. Specifically, 90-day flexural and compressive strengths with a 12.0-hour treatment duration show a 52.6% and 38.1% increase, respectively, compared to the control (without alkali-treated SDPs), as shown in S1 Table. This underscores the positive impact of 12.0-hour alkali treatment on surface etching, enhancing the bond strength between SDPs and the cementitious matrix.

In conclusion, a 12.0-hour alkali treatment of SDPs results in reduced fluidity and EC but increased flexural and compressive strengths. The dissolution of organic impurities and surface etching of SDPs emerge as pivotal factors influencing AAC performance. Consequently, careful consideration should be given to wastewater containing such organic impurities.

### Influence of sawdust wastewater on the properties

Sawdust, classified as a plant residue solid waste, undergoes various transformations such as decomposition, fermentation, heat generation, and odor emission during prolonged water immersion, a consequence of dissolved organic substances. As demonstrated in Figs 11–14, sawdust wastewater (SDW) has a substantial impact on AAC performance, prompting a more detailed examination of its effects on AAMs.

#### Fluidity and electrical conductivity

**Fig 15:**
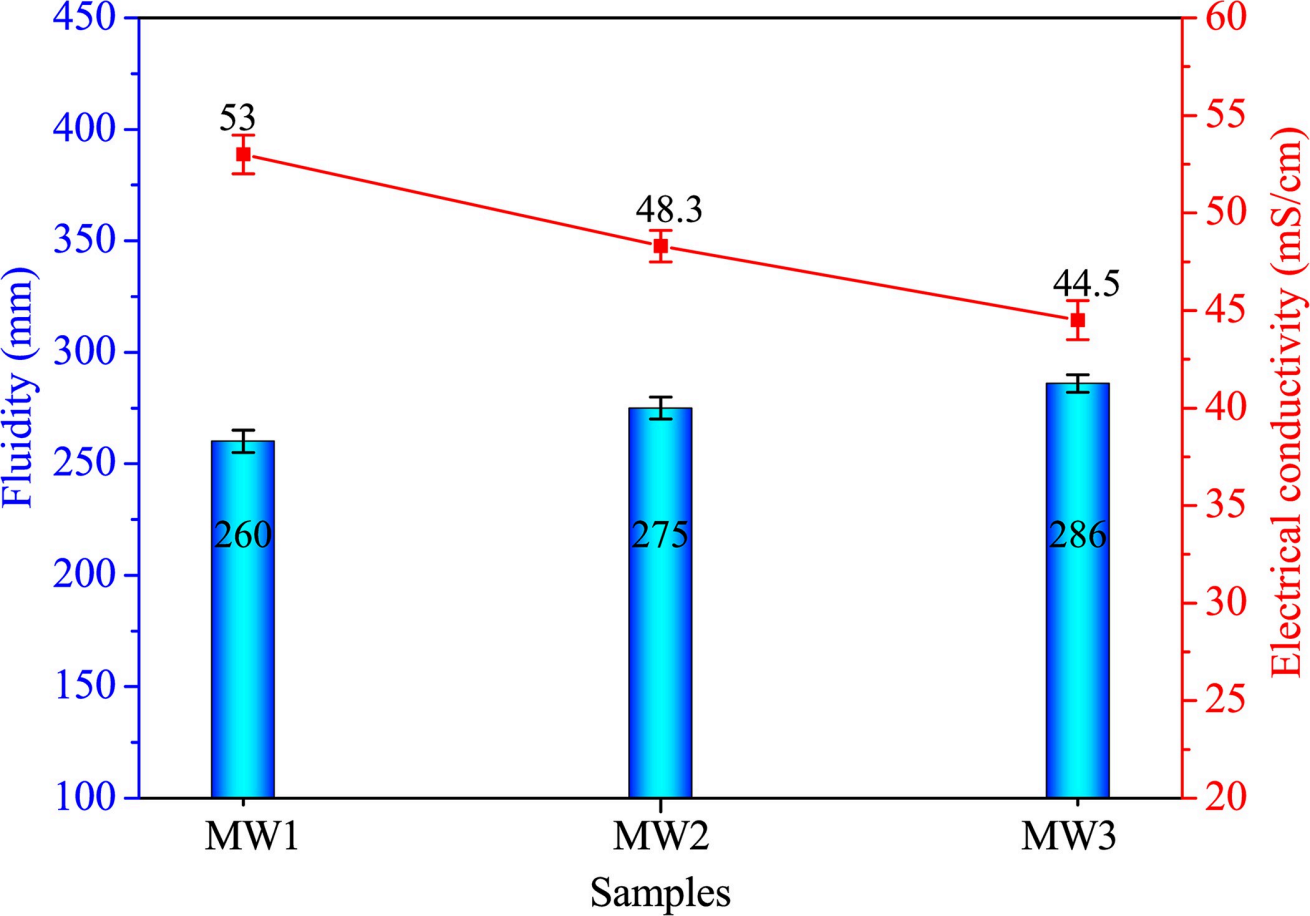
Fluidity and EC of alkali-activated composites with different SDW levels.

Fig 15 illustrates the outcomes of EC and fluidity for AACs incorporating different levels of SDW. The increase in SDW content (0.0% to 12.28%) leads to a decline in EC, while fluidity experiences a concurrent rise. Following water immersion treatment of SDPs, the resulting SDW contains an insignificant quantity of soluble conductive ions. Consequently, the introduction of SDW to the highly alkaline and conductive alkali-activated binder diminishes the EC of fresh AACs. The combination of low soluble ion concentration in SDW and increased water content contributes to the augmented fluidity in fresh AACs.

#### Drying shrinkage and density

**Fig 16:**
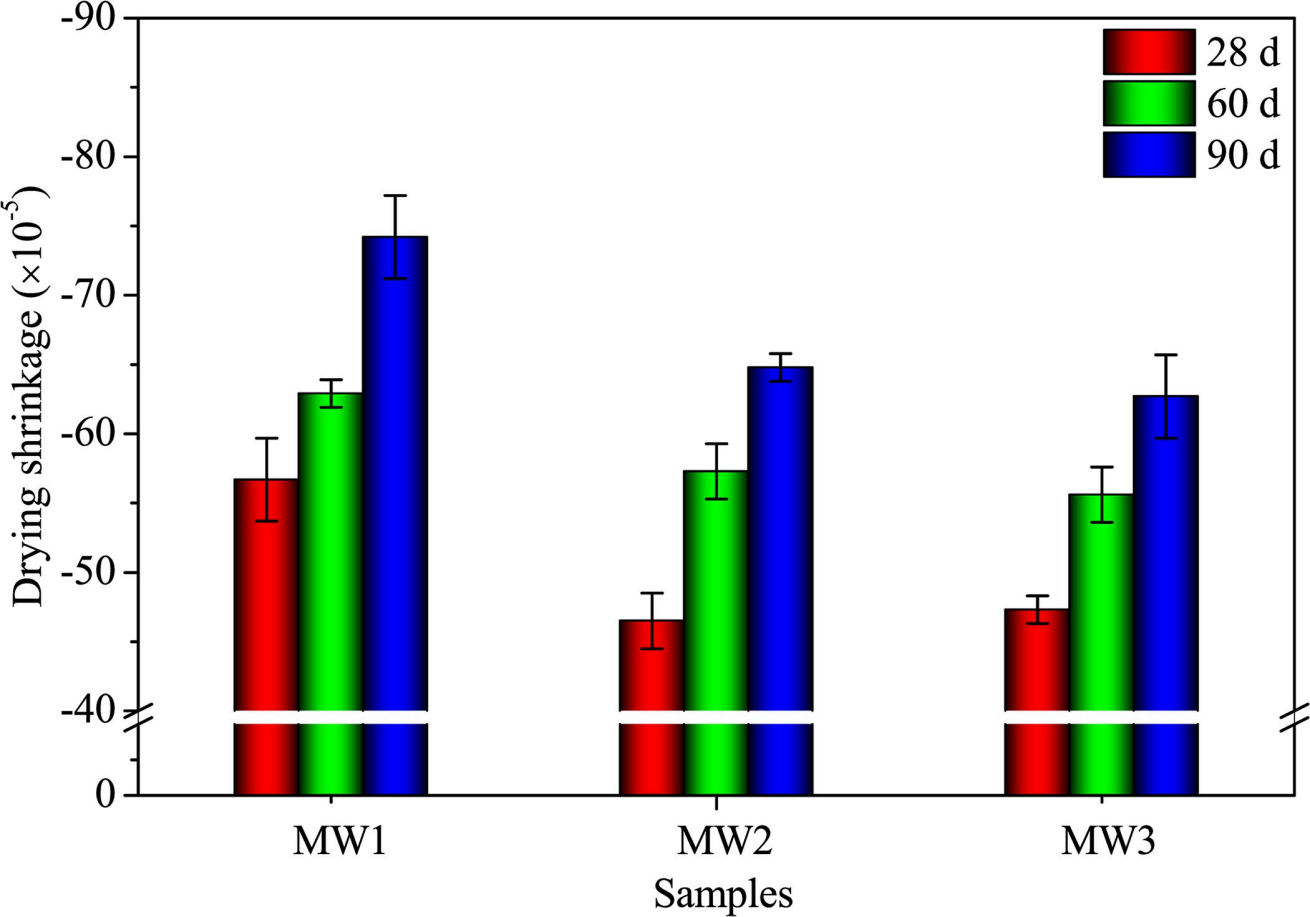
Drying shrinkage of alkali-activated composites with different SDW levels.

**Fig 17:**
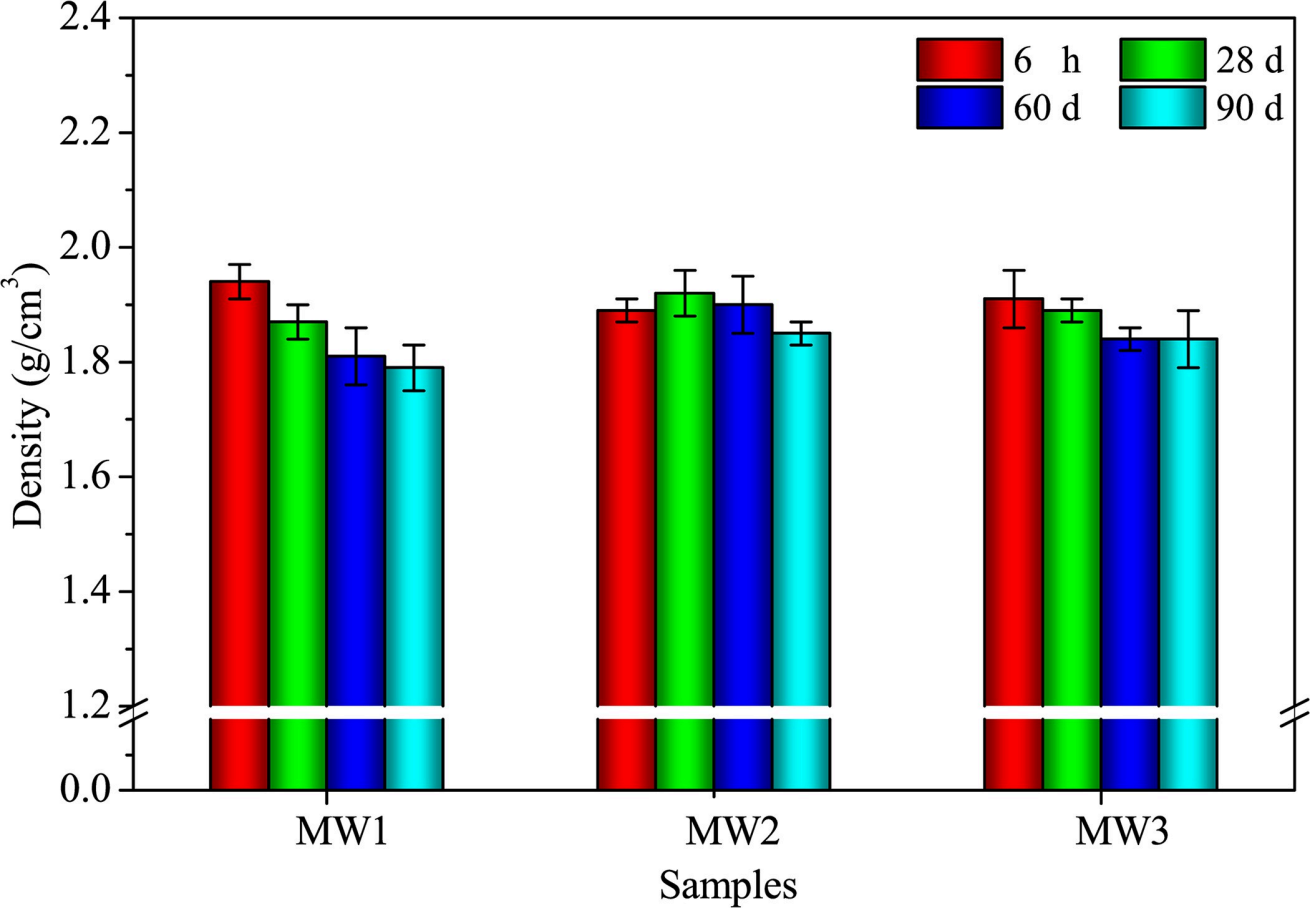
Density of alkali-activated composites with different SDW levels.

Fig 16 illustrates the drying shrinkages of DW1, DW2, and DW3 samples. Both samples show a gradual increase in drying shrinkage over the 90-day curing period. The rate of shrinkage from 28 to 90 days is smaller compared to the rate from 0 to 28 days (Fig 16). At the end of the 90-day curing period, DW1 has a drying shrinkage of 7.42×10⁻⁴, while DW3, which contains 12.28% SDW, exhibits a notable 15.50% reduction in drying shrinkage compared to DW1. The inclusion of SDW enhances the resistance against drying shrinkage in SP-RM-FA-based AAMs. The drying shrinkage in alkali-activated SP, RM, and FA is influenced by the physical and chemical properties of the materials, which traditionally demonstrate significant shrinkage [22, 44]. The addition of SDW introduces organic sodium salts, organic calcium salts, and the coexistence of inorganic silicates and aluminosilicates into the matrix, thereby improving the material’s shrinkage behavior [45]. The density of the samples decreases gradually as the curing age progresses, mainly due to water volatilization within the samples [45]. At 90 days, the addition of 12.28% SDW results in a modest 3.4% increase in density (Fig 17). In conclusion, SDW effectively reduces the drying shrinkage of AACs.

#### Flexural and compressive strengths

**Fig 18:**
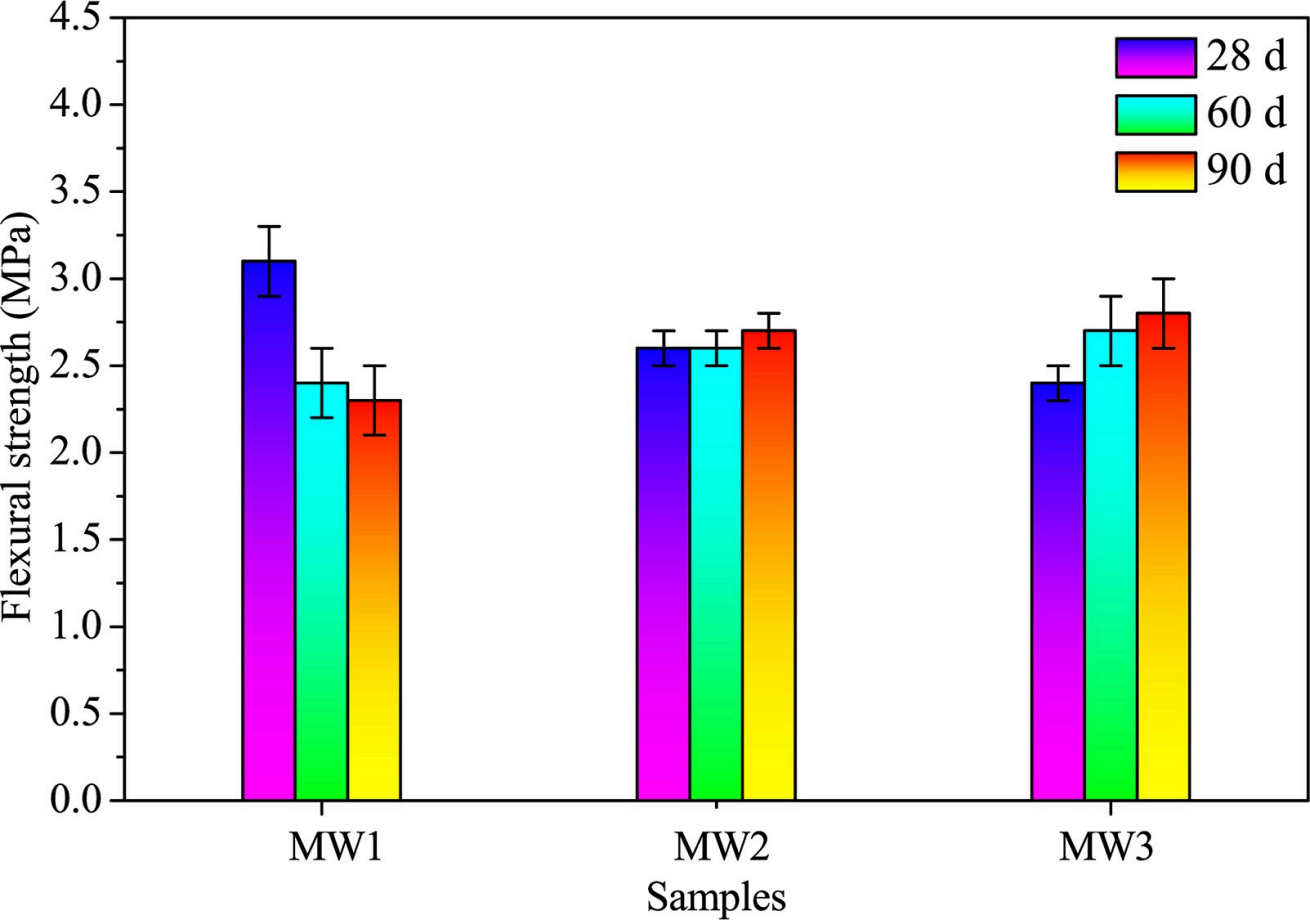
Flexural strengths of alkali-activated composites with different SDW levels when cured for 28, 60, and 90 days.

**Fig 19:**
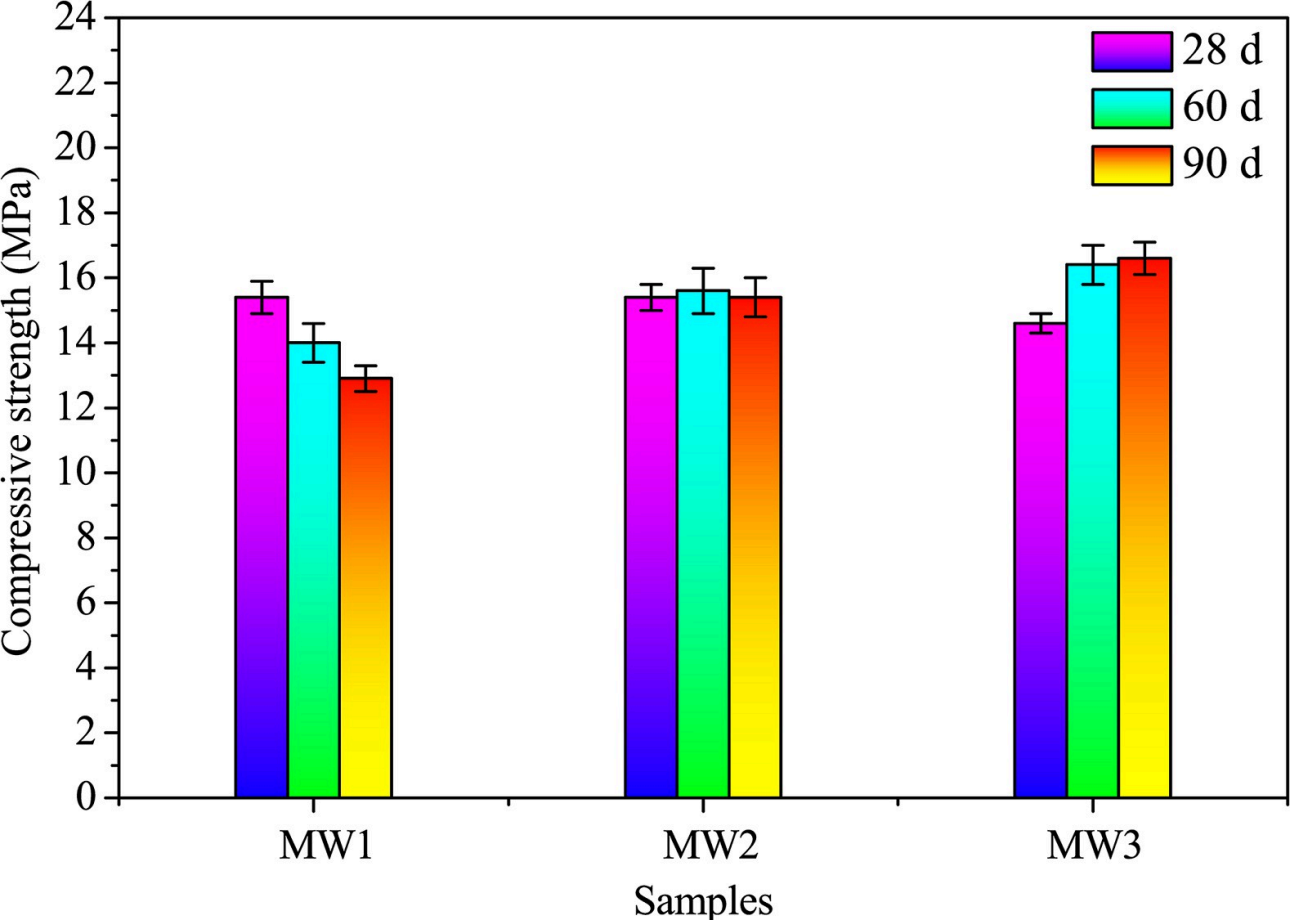
Compressive strengths of alkali-activated composites with different SDW levels when cured for 28, 60, and 90 days.

Figs 18 and 19 illustrates the flexural and compressive strengths of AACs at 28, 60, and 90 days, respectively. The sample MW1, devoid of SDW, undergoes rapid solidification, achieving flexural strengths of 3.1 MPa and compressive strengths of 15.4 MPa at 28 days. However, a subsequent decrease in both flexural and compressive strengths is noted from 28 to 90 days, resulting in reductions of 25.81% and 16.23%, respectively, compared to 28 days. This decline is attributed to cracks and alkali-activation product formation within the SP-RM-FA system. The initial mixing stages involve alkaline solution dissolution of the silica-aluminate phase, leading to polymerization and hydration reactions with CaO and NaOH, forming calcium-containing silica-aluminate gel and silicate gel [46, 47]. While these gels enhance the system’s binding strength, the sustained dissolution and polymerization diminish over time due to lower NaOH concentration (2.5 mol/L) and moisture loss, resulting in a slight polymer strength decline rather than a continuous increase.

The introduction of SDW (ranging from 0.00% to 12.28%) causes an initial decrease in flexural and compressive strengths at 28 days but leads to an increase at 90 days. Sample MW3, featuring 12.28% SDW, initially exhibits lower flexural and compressive strengths at 28 days compared to MW1. However, these strengths progressively increase with aging. At 90 days, MW3 demonstrates a 21.74% increase in flexural strength and a 28.68% increase in compressive strength compared to MW1 (as shown in S2 Table). This positive impact on long-term mechanical strength can be attributed to the decomposition and degradation of SDPs upon water immersion, releasing organic substances like pectin, organic acids, sugars, and hemicellulose into the water, rendering SDW acidic [18]. These organics react with alkaline substances (NaOH and CaO), forming organic acid sodium or organic acid calcium [45]. Additionally, AAMs exhibit the capacity to adsorb harmful substances [48, 49]. In an alkaline environment, organic acid calcium aids in the hydration process of the silicon-aluminum-calcium-sodium multicomponent system, augmenting binding properties in later stages [45]. Consequently, further microscopic investigations are undertaken to elucidate the mechanistic effect of SDW on AACs.

### Influence of sawdust wastewater on the microstructures and products

#### Mineral compositions analysis by XRD

**Fig 20:**
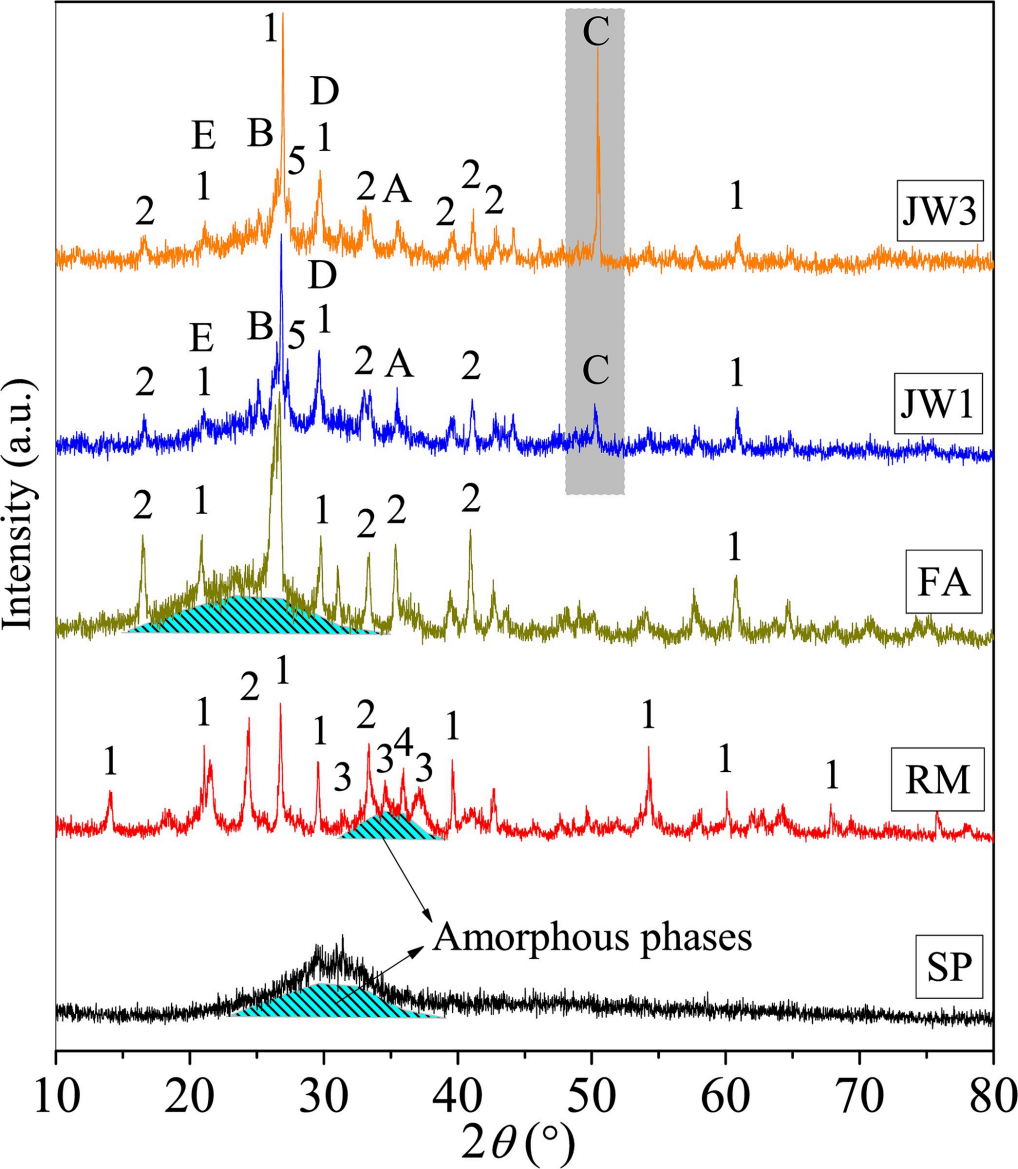
XRD patterns of alkali-activated samples (JW1 and JW3) and raw materials (SP, RM, and FA).

Fig 20 presents the XRD spectra of 90-day samples JW1 and JW3, as well as those of raw materials (SP, RM, and FA). The XRD patterns indicate that the SP predominantly comprises amorphous phases, manifested by broad non-diffracting humps observed within the 22˚ to 40˚2θ range. Conversely, FA is primarily constituted of crystalline phases, including Quartz and Mullite, along with amorphous phases rich in silicon and aluminum, which are evidenced by the humps within the 15˚ to 35˚2θ range. Similarly, RM is composed primarily of crystalline phases, such as Quartz, Mullite, hydrated calcium aluminate (C-A-H), hydrated calcium silicate (C-S-H), and partial amorphous phases rich in silicon and aluminum, as denoted by humps within the 30˚ to 40˚2θ range.

A noticeable distinction from raw materials is the formation of new mineral phases in the alkali-activated sample at 90-d age, including sodium alumino-silicate (N-A-S-H) zeolite, C-S-H, calcium hydroxide (Ca(OH)_2_), calcium carbonate (CaCO_3_), and two types of hydrated calcium-aluminum-silicate (C-A-S-H) gels (AS and AS_2_), as shown in Table 3. However, the diffraction peaks of C-S-H and C-A-H in the RM are diminished due to their low proportions resulting from mixing reactions or encapsulation within the gel products. Remarkably, the introduction of SDW leads to similar mineral phase formation in JW3 as observed in JW1, with only slight changes in the intensity of the diffraction peaks related to the CaCO_3_ near 50˚. These observations indicate that the mineral compositions of AAMs remain unaffected by SDW. Notably, the formation of N-A-S-H zeolite is attributed to the crystallization of the amorphous N-A-S-H polymer gel induced by alkali activation in the long term. C-S-H and C-A-S-H originate partially from the RM, primarily as a result of hydration reactions involving CaO, SiO_2_, Al_2_O_3_, and H_2_O. However, certain amorphous substances, such as amorphous N-A-S-H gel, C-S-H gel, and C-A-S-H gel, are detected by XRD patterns, yet correspond to the humps in the range of 22˚ to 36˚2θ. Under alkaline conditions, the crystallization of Ca(OH)_2_ occurs. However, over an extended curing period, carbonation reactions between CO_2_ in the air and Ca(OH)_2_ give rise to the formation of CaCO_3_ crystals. Consequently, during a 90-day curing age, excluding the carbonation, the incorporation of 12.28% SDW does not alter the mineral compositions of SP-RM-FA-based AACs. The AACs retain their characteristic amorphous N-A-S-H, C-S-H, and C-A-S-H gels, coexisting with crystalline phases, thus contributing to the formation of a solidified matrix.

**Table 3:** The mineral compositions detected by the XRD method in Fig 20.

No.	Mineral Phases (pdf# card number)	No.	Mineral Phases (pdf# card number)
**1**	Quartz (pdf# 97-003-9830)	**A**	Ca(OH)_2_ (pdf# 00-003-0865)
**2**	Mullite (pdf# 00-029-1487)	**B**	N-A-S-H (pdf# 00-056-0499)
**3**	C-A-H (pdf# 97-041-8966)	**C**	CaCO_3_ (pdf# 99-000-4172)
**4**	C-S-H (pdf# 97-024-0406)	**D**	C-A-S-H (pdf# 99-000-4228)
**5**	C-S-H (pdf# 97-034-0002)	**E**	C-A-S-H (pdf# 00-015-0179)

#### Chemical bonds analysis by FTIR

**Fig 21:**
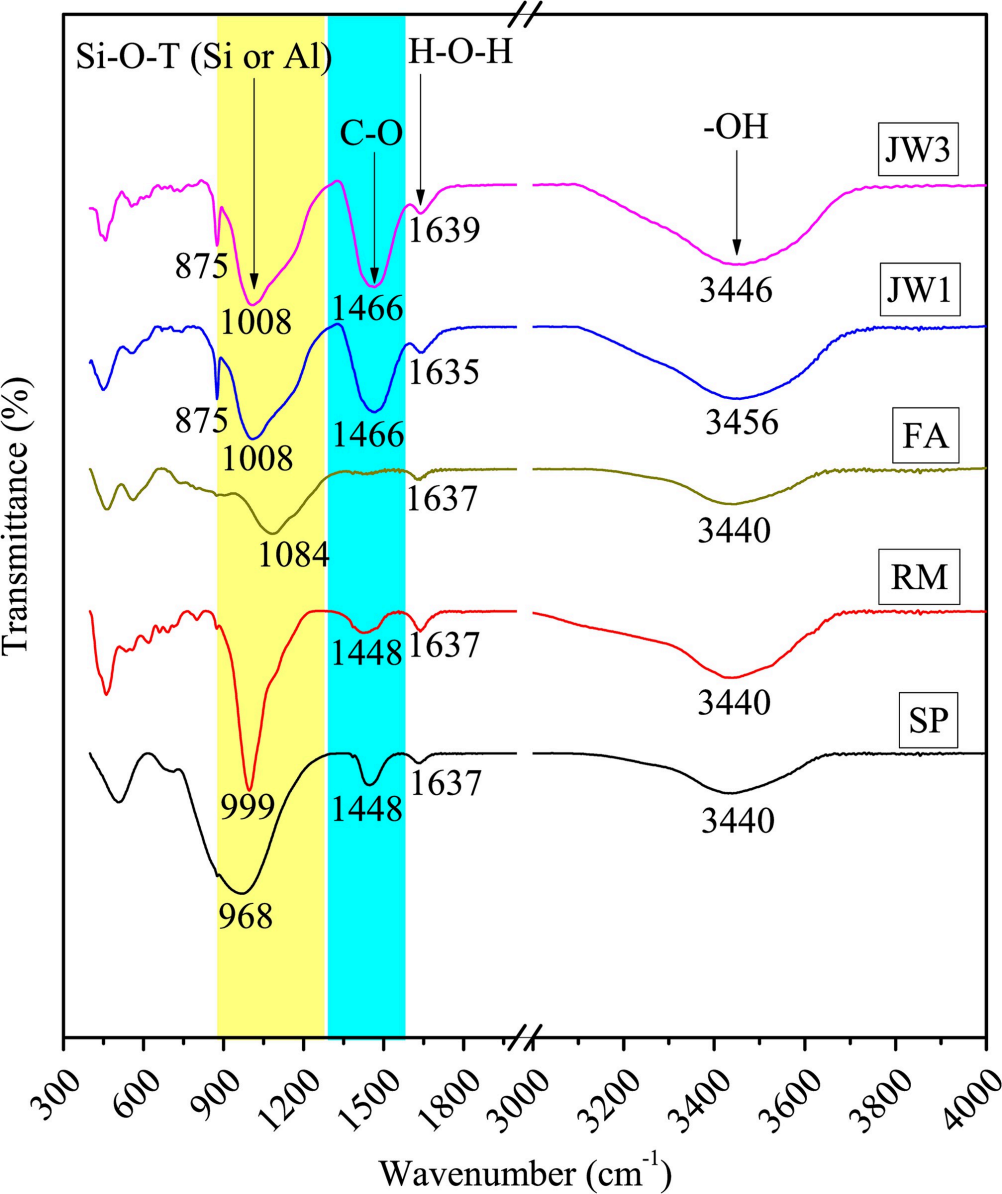
FTIR spectra of alkali-activated samples (JW1 and JW3) and raw materials (SP, RM, and FA).

Fig 21 shows the FTIR spectra of 90-day samples JW1 and JW3, as well as the raw materials. In the raw material, there is no absorption peak at 875 cm^-1^, but this peak is present in JW1 and JW3, indicating CO_3_^2-^ bending vibrations [50] and carbonate formation in alkali-activated SP-RM-FA materials [51, 52]. The strong absorption peaks at 1448 cm^-1^ and 1466 cm^-1^ represent stretching and bending vibrations of the C-O bond [53], indicating increased carbonization in JW1 and JW3, which is consistent with XRD measurements in Fig 20. The peak at 1008 cm^-1^ corresponds to the asymmetric stretching vibration of Si-O-T (Si or Al) bonds, which shifts due to NaOH-induced Si-O-Si chain formation in SP [54], and Si-O-Al chain formation in FA. This favors the generation of C-S-H products and silica-aluminate polymer formation [55]. JW1 shows the coexistence of silicates and silica-aluminates, as seen in previous studies [46, 56].

The peaks at 1635 cm^-1^, 1637 cm^-1^, and 1639 cm^-1^ represent H-O-H bond bending vibrations in water molecules [57] The peaks at 3440 cm^-1^, 3446 cm^-1^, and 3456 cm^-1^ represent stretching vibrations of hydroxyl groups (-OH) in the raw material, product structure, and cellulose molecules [58]. Comparing JW1 and JW3, shifts in wavenumbers indicate variations in water molecule association. Notably, JW3’s FTIR spectra do not show characteristic peaks in the range of 2700–2900 cm^-1^ [24, 59] associated with fatty components in cellulose, hemicellulose, and lignin. This confirms that 12.28% SDW does not alter product compositions in SP-RM-FA-based AACs. Organic impurities in SDW are trapped in the alkali-activated matrix, and organic acid salts generated react, decompose, and disappear during prolonged room temperature curing. Therefore, the effect mechanism of SDW on product gels is chemical in nature, including the carbonation.

#### Morphological and elemental analysis by SEM and EDS

**Fig 22:**
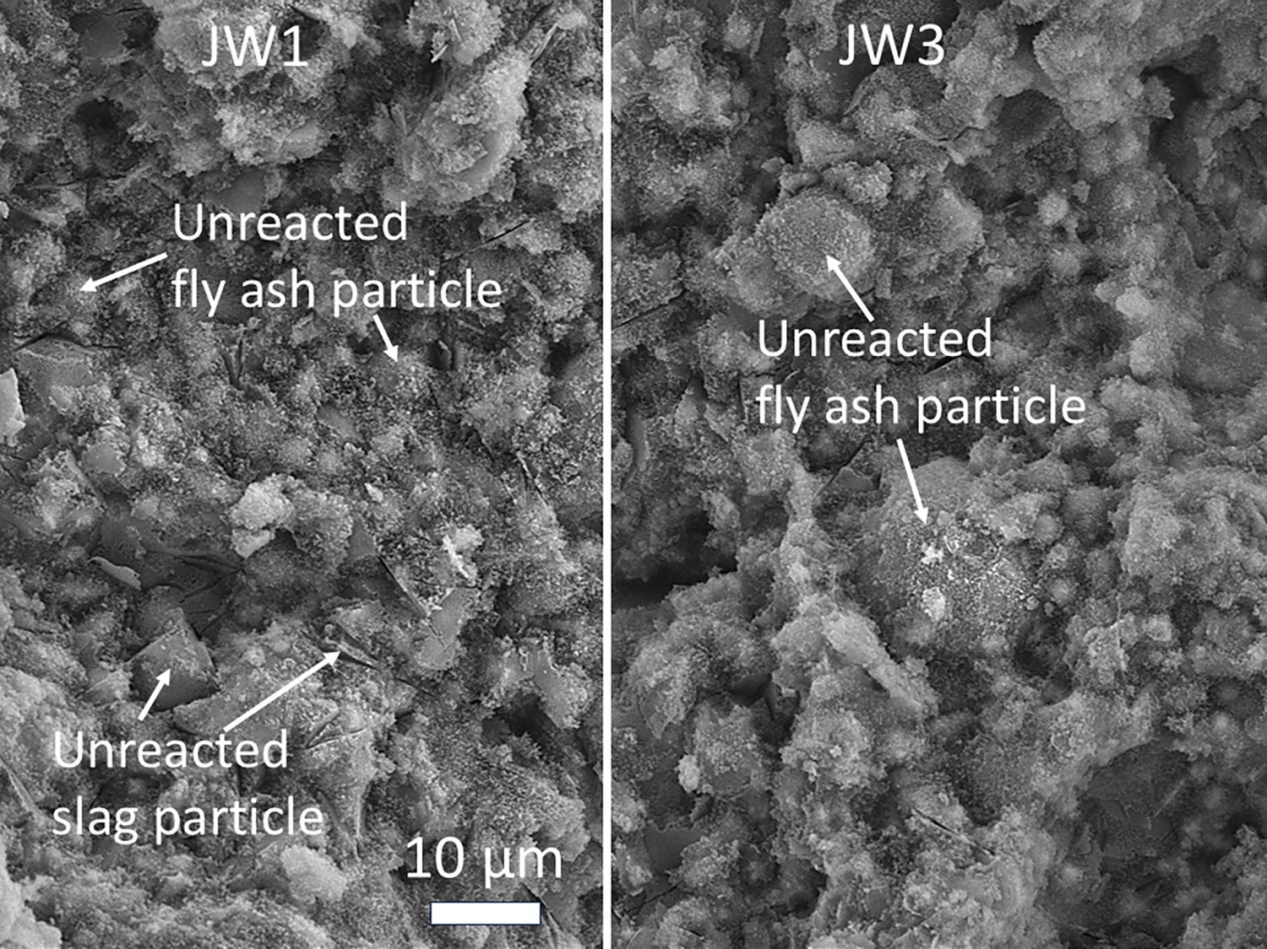
SEM images of alkali-activated samples JW1 and JW3 at a magnification of ×1000.

**Fig 23:**
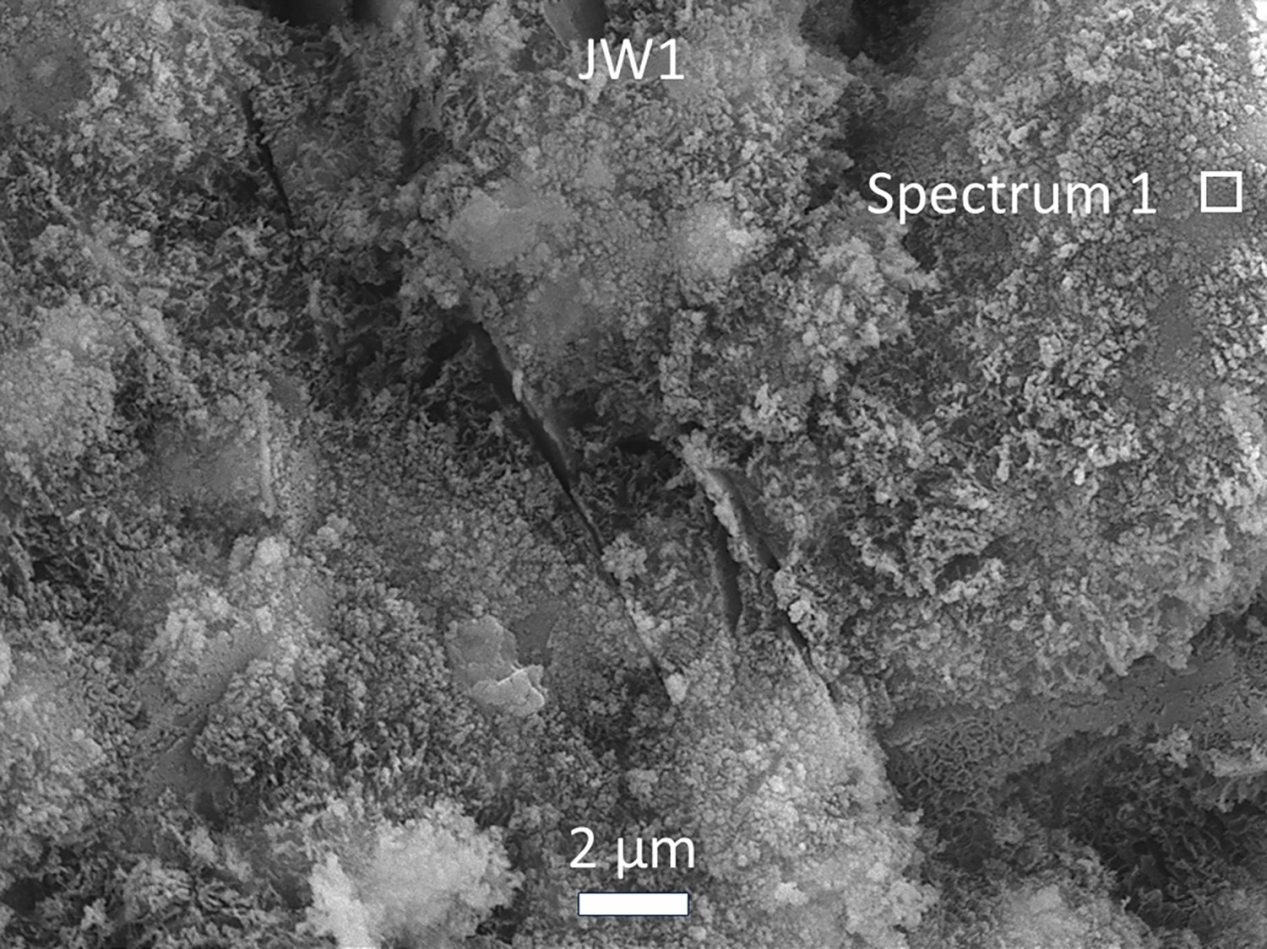
SEM images of alkali-activated samples JW1 at a magnification of ×5000.

**Fig 24:**
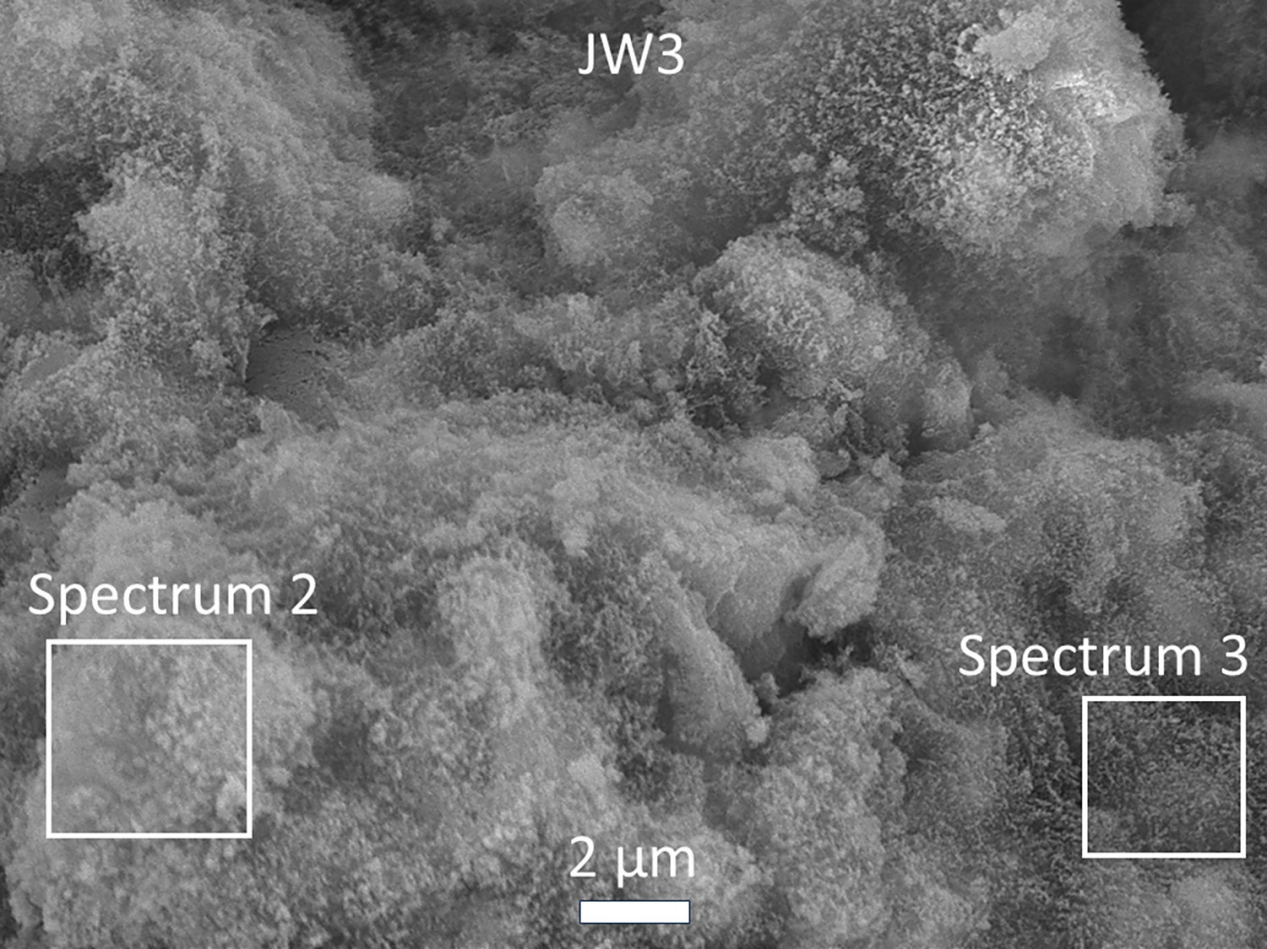
SEM images of alkali-activated samples JW3 at a magnification of ×5000.

**Fig 25:**
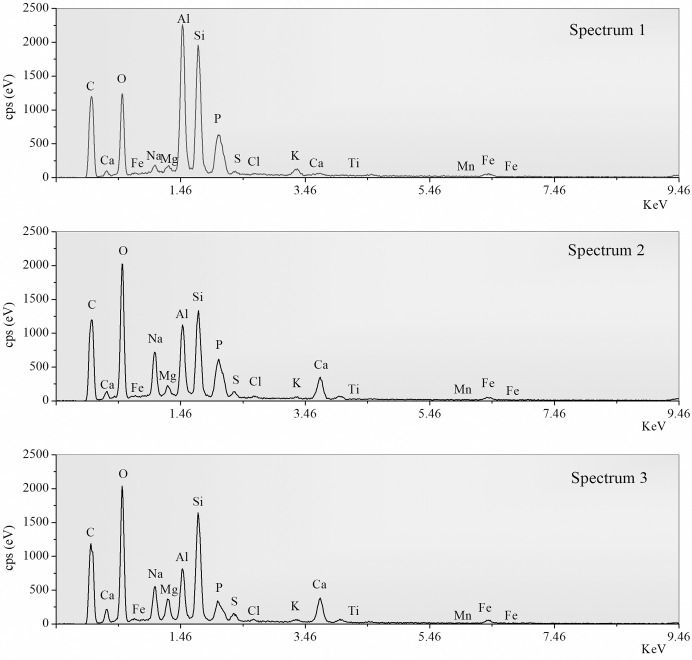
EDS spectra from the detected areas in samples JW1 and JW3.

Microstructural images in Figs 22–25 depict fractured surfaces of samples JW1 and JW3 at 90 days. In Fig 22, the binder phase morphology of JW1 shows irregular unreacted SP and spherical unreacted FA particles exposed on the fractured surface. These particles, alongside chemically formed gel, create a supportive framework. Fig 22 reveals JW3, incorporating 12.23% SDW, with enhanced consolidation and denser structure compared to JW1. JW3 still contains unreacted FA particles, but a notable reduction in irregular unreacted SP particles is evident (Figs 23 and 24).

Elemental composition analysis of JW1 and JW3 indicates primary elements as C, Ca, O, Fe, Na, Mg, Al, and Si in representative regions (Fig 25 and Table 4). Spectrum 3 shows higher C content, while Spectrum 2 exhibits lower C content compared to Spectrum 1, suggesting potential uneven carbonation in AACs with SDW. The EDX spectra from points 2 and 3 in the SEM image show differences in the C, Al, Na, and P elements, which is due to the heterogeneity of the sample. The purpose of using two points is also to mitigate the effects of this heterogeneity. The reason for the larger difference in the C element between the two points may be attributed to variations in the local carbonization degree of the sample. Combining FTIR and XRD results, C elements are not persistent organic impurities but result from the reaction of alkaline Ca(OH)_2_ and NaOH on the fractured surface with CO_2_, forming carbonates. Consequently, weight percentages of O, Ca, and K elevate, while Si and Al contents reduce (as shown in S2–S5 Figs). This suggests that incorporating 12.23% SDW exposes NaOH content on the fractured surface in AACs.

**Table 4:** Elemental compositions detected by the EDS method corresponding to Fig 25.

Spectrum 1 (Sample JW1)	Wt.%	Spectrum 2 (Sample JW3)	Wt.%	Spectrum 3 (Sample JW3)	Wt.%
**C**	11.20	C	9.42	C	16.33
**O**	34.35	O	45.65	O	43.55
**Na**	1.24	Na	7.92	Na	5.17
**Mg**	0.83	Mg	1.35	Mg	2.68
**Al**	20.55	Al	9.66	Al	6.10
**Si**	22.54	Si	12.82	Si	13.61
**P**	1.36	P	1.11	P	0.47
**S**	0.83	S	1.37	S	1.40
**Cl**	0.15	Cl	0.32	Cl	0.37
**K**	2.07	K	0.38	K	0.50
**Ca**	0.80	Ca	6.78	Ca	6.52
**Ti**	0.64	Ti	0.26	Ti	0.37
**Mn**	0.09	Mn	0.00	Mn	0.00
**Fe**	3.39	Fe	2.95	Fe	2.94
**Total:**	100.00	Total:	100.00	Total:	100.00

## Conclusions

The objective of this study was to evaluate the feasibility of incorporating sawdust particles (SDPs) and sawdust wastewater (SDW) in alkali-activated solid waste-based composites, specifically alkali-activated composites (AACs). The research focused on the effects of SDP content, duration of treatment for SDP, and SDW content on the properties of fresh and hardened AACs. The study yielded significant findings:

(1) Increasing the concentration of SDP in AACs reduces their fluidity and electrical conductivity (EC). However, treating SDPs with a 2.5 mol/L NaOH solution for 12.0 hours results in decreased fluidity and EC, but increased flexural and compressive strengths. This indicates that appropriate alkali treatment of SDPs positively affects the mechanical characteristics of AACs.

(2) In the composite material synthesis using binder materials SP, RM, FA, CS in a mass ratio of 2:2:3:2, mixed with a 2.0 mol/L Na_2_SiO_3_ solution, with a liquid-to-solid ratio of 11:9 and a sand-to-binder ratio of 3:1, the SDP content is 10% (sawdust particles account for the mass of the binder materials). The SDPs are soaked in a 2.5 mol/L NaOH solution for 12.0 hours compared to the control group, which help to synthesize a composite material that improves flexural and compressive strengths.

(3) Incorporating 12.28% SDW is beneficial for reducing drying shrinkage and promoting long-term mechanical strength development in AACs based on SP-RM-FA. The mineral compositions and microchemical bonds of AACs remain unchanged over a 90-day curing period. The presence of amorphous N-A-S-H, C-S-H, and C-A-S-H gels, along with crystalline phases, contributes to a solidified matrix.

(4) In the composite material synthesis using binder materials SP, RM, FA in a mass ratio of 10:3:18, mixed with a 2.0 mol/L NaOH solution, with a liquid-to-solid ratio of 20:31 and a sand-to-binder ratio of 3:1, SDW replaces 12.28% of the sodium hydroxide solution. The SDPs are soaked in water at a 1:20 mass ratio for 3 days, then filtered to obtain the SDW. The composite material obtained in this way shows a significant increase in flexural and compressive strengths compared to the control group, with a significant enhancement in post-curing strength. Acidic SDW introduces organic impurities into the alkali-activated solid waste-based matrix. Organic acid salts are formed when these impurities come into contact with alkaline CaO or NaOH.

Overall, this study promotes the use of alkali-activated solid waste-based products, including SDPs and SDW, to achieve cleaner manufacturing. It provides valuable empirical information on the impact of sawdust and wastewater on AACs, contributing to the development of cleaner materials. The integration of industrial and agricultural solid wastes-wastewater presents an innovative approach that promotes the production of eco-friendly, low-emission products. Further research is needed to investigate the variability of wastewater from different facilities, allowing for the development of customized construction materials for various applications.

## Supporting information

S1 Fig The SEM images of sawdust particles (SDPs) in sample MS4 with 10.0% SDPs. (ZIP)

S2 Fig SEM images of alkali-activated samples JW1 and JW3 at a magnification of ×1000. (ZIP)

S3 Fig SEM images of alkali-activated samples JW1 at a magnification of ×5000. (ZIP)

S4 Fig SEM images of alkali-activated samples JW3 at a magnification of ×5000. (ZIP)

S5 Fig EDS spectra from the detected areas in samples JW1 and JW3. (ZIP)

S1 Table Compressive strengths of samples with different alkali-treated durations of SDPs. (ZIP)

S2 Table Compressive strengths of samples with different SDW levels. (ZIP)
