# Supplementary figures and images for: Properties characterization and microstructural analysis of alkali-activated solid waste-based materials with sawdust and wastewater integration
Source: PLoS One. 2025 Jan 3;20(1):e0313413. doi: 10.1371/journal.pone.0313413 (PMC11698524; doi:10.1371/journal.pone.0313413)

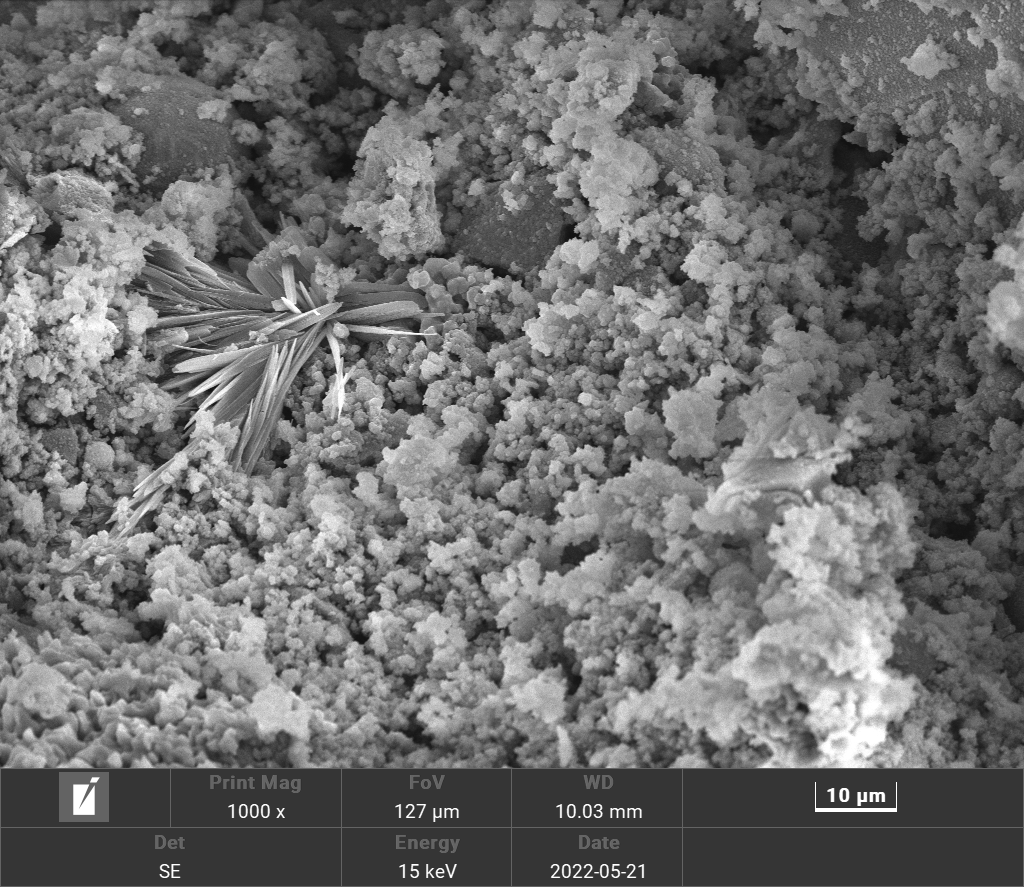

Supplement: S1 Fig — (ZIP) [file pone.0313413.s001.zip › S1_Fig/Fig 10-(Left).TIF]

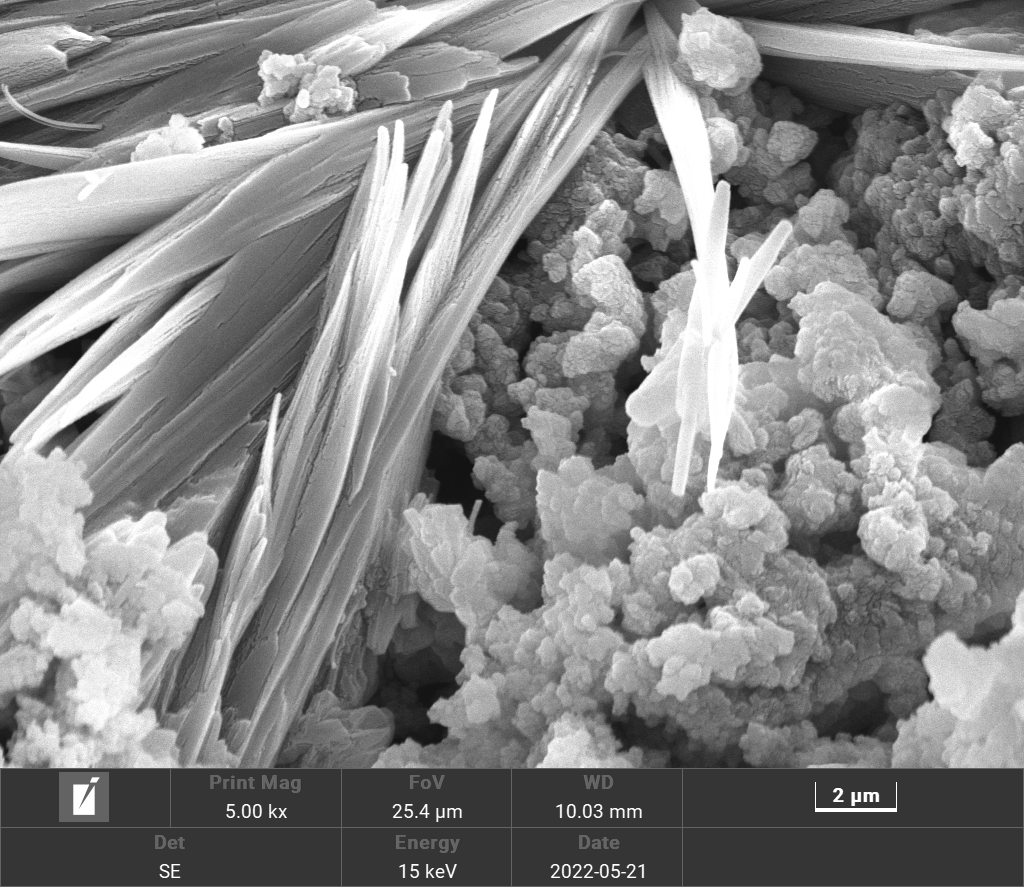

Supplement: S1 Fig — (ZIP) [file pone.0313413.s001.zip › S1_Fig/Fig 10-(Right).TIF]

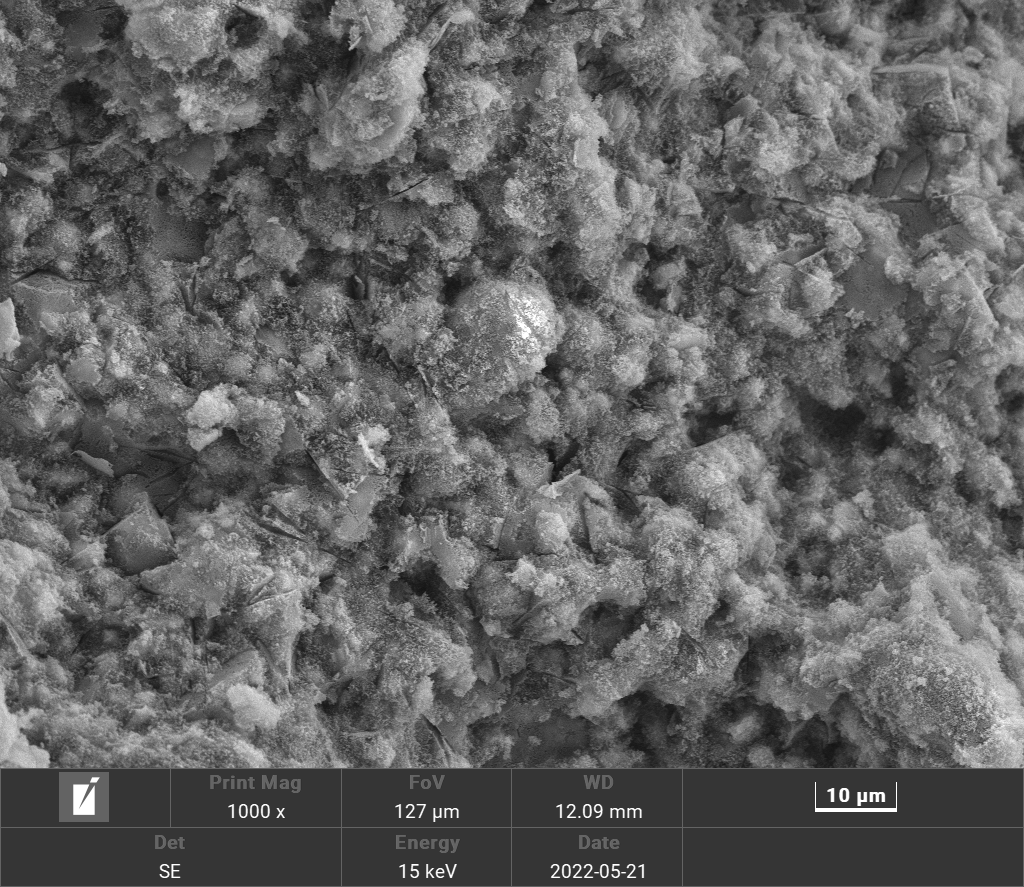

Supplement: S2 Fig — (ZIP) [file pone.0313413.s002.zip › S2_Fig/Fig 22-(Left).TIF]

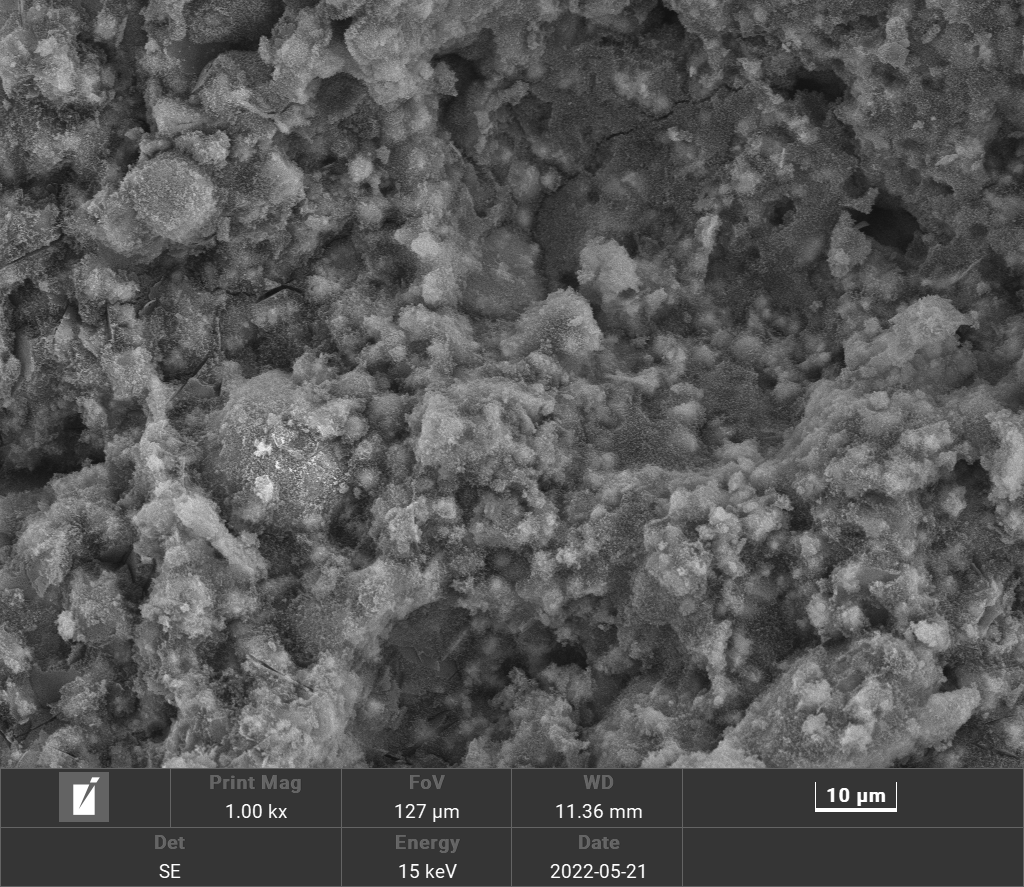

Supplement: S2 Fig — (ZIP) [file pone.0313413.s002.zip › S2_Fig/Fig 22-(Right).TIF]

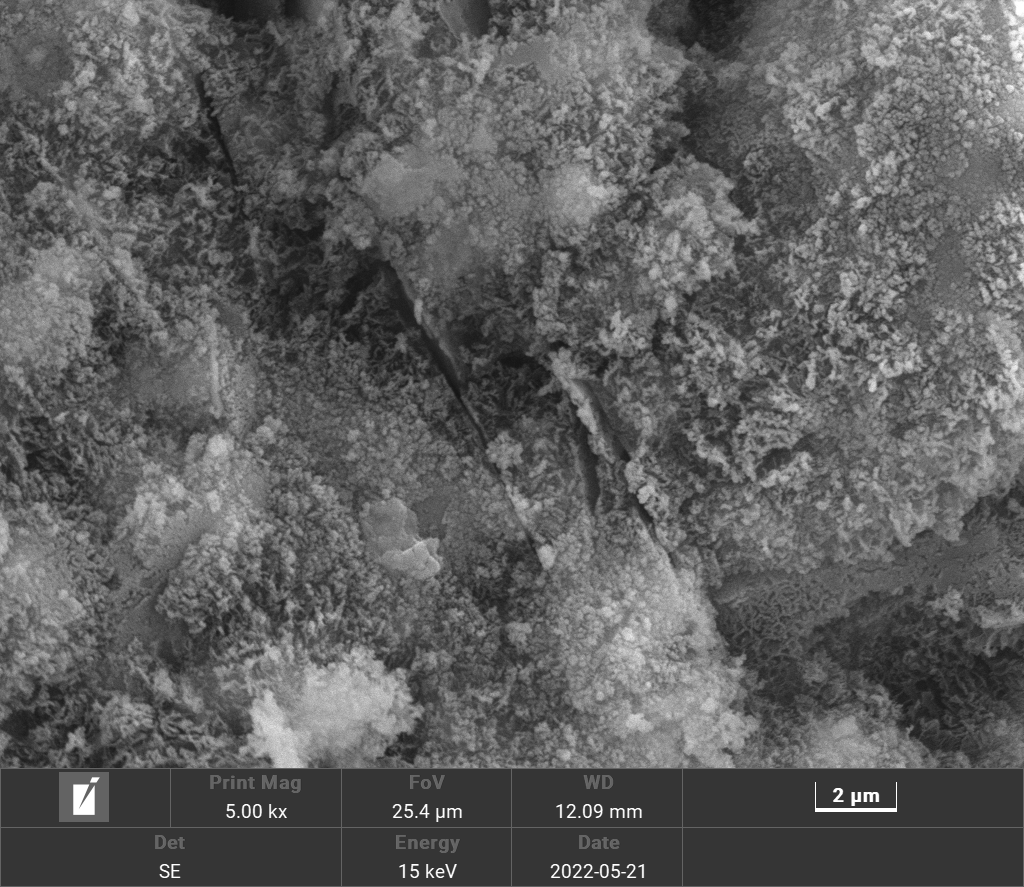

Supplement: S3 Fig — (ZIP) [file pone.0313413.s003.zip › S3_Fig/Fig 23.TIF]

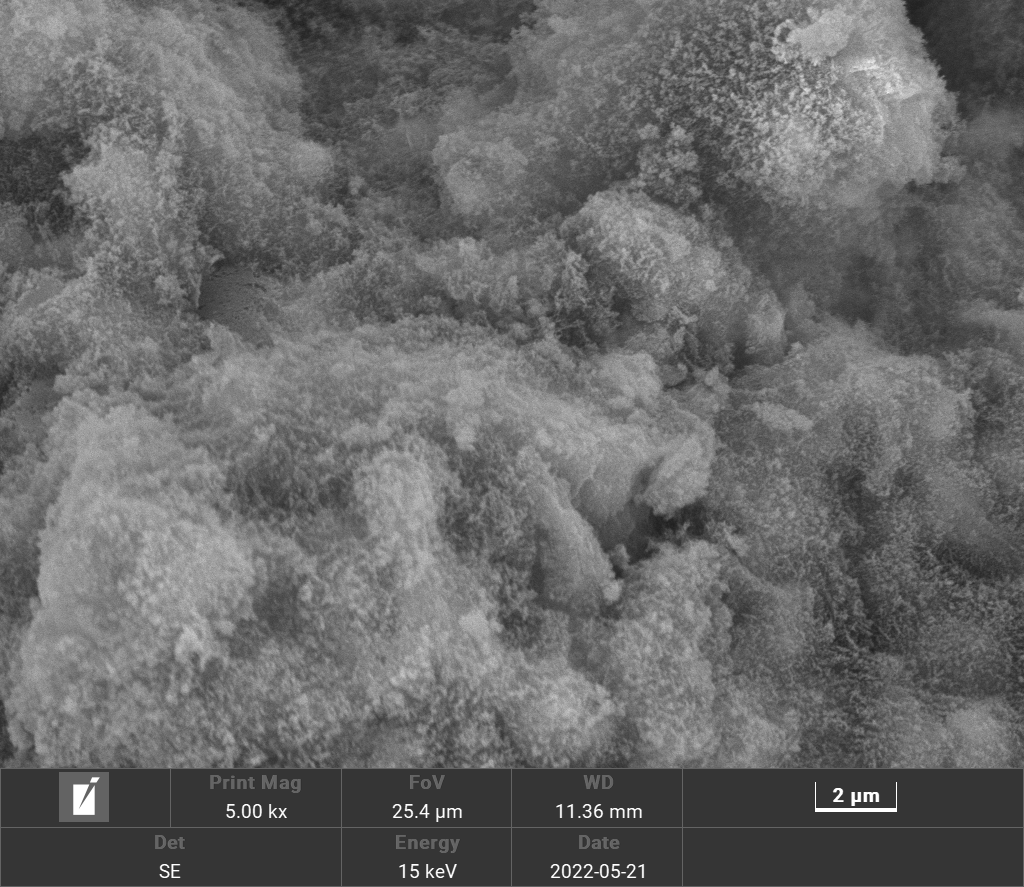

Supplement: S4 Fig — (ZIP) [file pone.0313413.s004.zip › S4_Fig/Fig 24.TIF]
