# Supplementary material for: Properties characterization and microstructural analysis of alkali-activated solid waste-based materials with sawdust and wastewater integration
Source: PLoS One. 2025 Jan 3;20(1):e0313413. doi: 10.1371/journal.pone.0313413 (PMC11698524; doi:10.1371/journal.pone.0313413)
Supplement: S5 Fig — (ZIP) [file pone.0313413.s005.zip › S5_Fig/Fig 25 (down).docx]

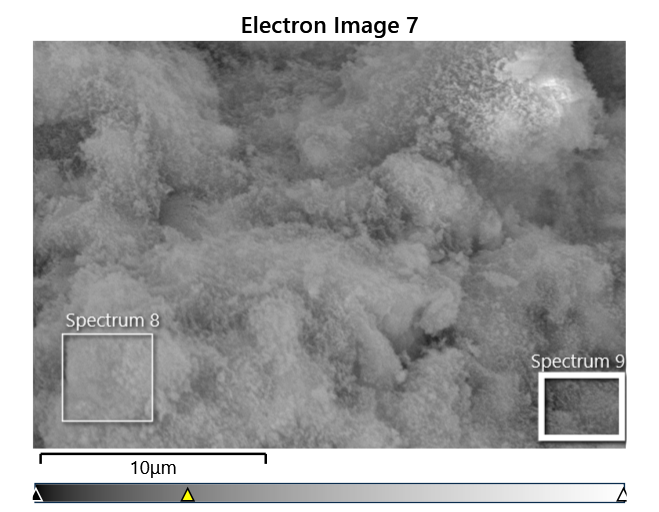

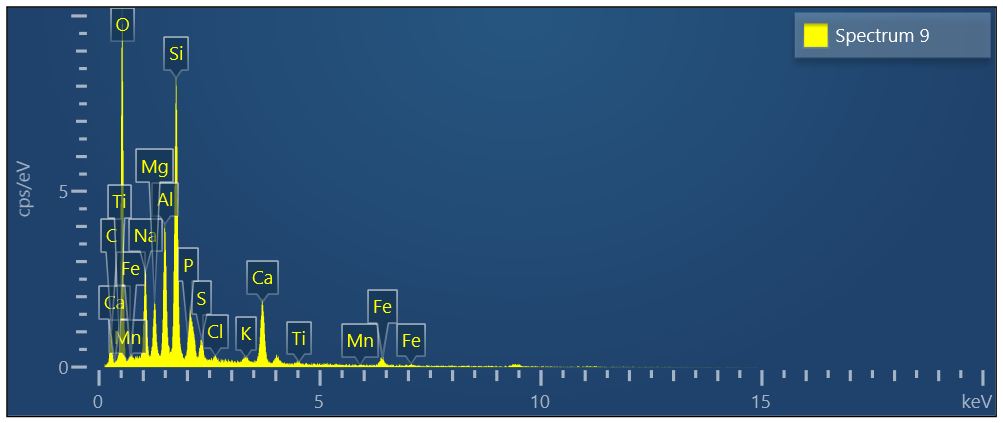


| **Spectrum 9** |  |  |  |  |  |  |  |  |
| --- | --- | --- | --- | --- | --- | --- | --- | --- |
| Element | Line Type | Apparent Concentration | k Ratio | Wt% | Wt% Sigma | Standard Label | Factory Standard | Standard Calibration Date |
| C | K series | 4.12 | 0.04125 | 16.33 | 1.36 | C Vit | Yes |  |
| O | K series | 53.24 | 0.17917 | 43.55 | 0.84 | SiO2 | Yes |  |
| Na | K series | 7.39 | 0.03117 | 5.17 | 0.19 | Albite | Yes |  |
| Mg | K series | 3.02 | 0.02002 | 2.68 | 0.13 | MgO | Yes |  |
| Al | K series | 7.14 | 0.05132 | 6.10 | 0.18 | Al2O3 | Yes |  |
| Si | K series | 15.82 | 0.12534 | 13.61 | 0.31 | SiO2 | Yes |  |
| P | K series | 0.75 | 0.00417 | 0.47 | 0.13 | GaP | Yes |  |
| S | K series | 1.58 | 0.01365 | 1.40 | 0.10 | FeS2 | Yes |  |
| Cl | K series | 0.42 | 0.00367 | 0.37 | 0.08 | NaCl | Yes |  |
| K | K series | 0.62 | 0.00528 | 0.50 | 0.09 | KBr | Yes |  |
| Ca | K series | 7.92 | 0.07079 | 6.52 | 0.21 | Wollastonite | Yes |  |
| Ti | K series | 0.37 | 0.00366 | 0.37 | 0.12 | Ti | Yes |  |
| Mn | K series | 0.00 | 0.00000 | 0.00 | 0.16 | Mn | Yes |  |
| Fe | K series | 2.91 | 0.02906 | 2.94 | 0.27 | Fe | Yes |  |
| Total: |  |  |  | 100.00 |  |  |  |  |
