# Supplementary material for: Properties characterization and microstructural analysis of alkali-activated solid waste-based materials with sawdust and wastewater integration
Source: PLoS One. 2025 Jan 3;20(1):e0313413. doi: 10.1371/journal.pone.0313413 (PMC11698524; doi:10.1371/journal.pone.0313413)
Supplement: S5 Fig — (ZIP) [file pone.0313413.s005.zip › S5_Fig/Fig 25 (middle).docx]

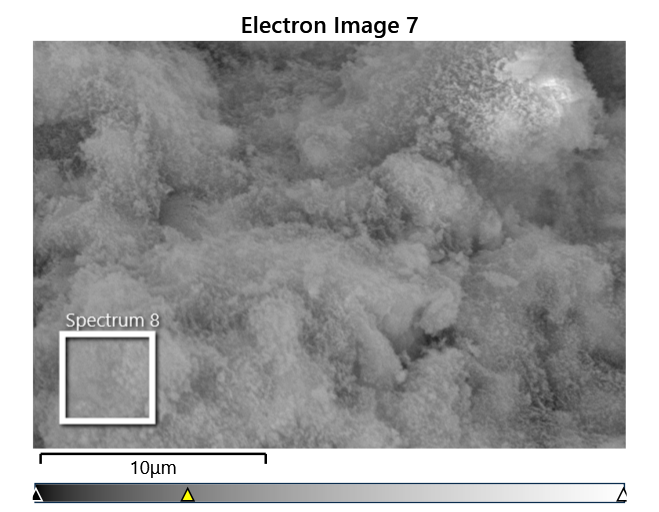

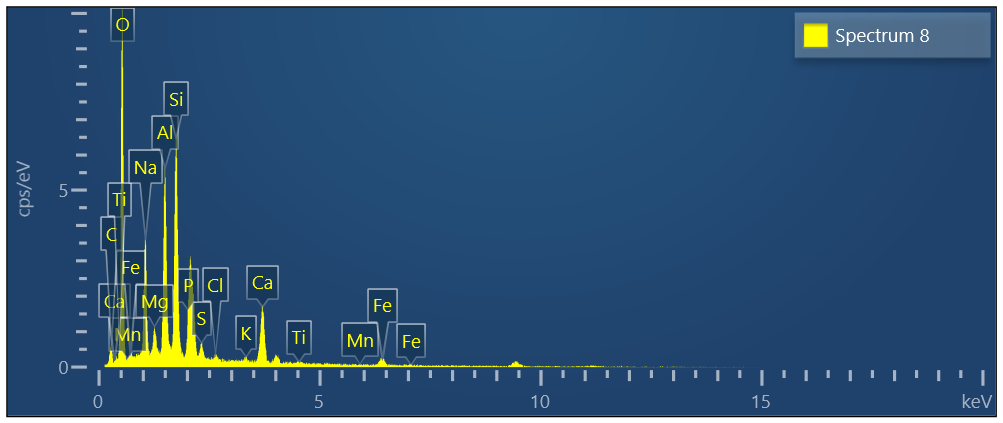


| **Spectrum 8** | | | | | | | | |
| --- | --- | --- | --- | --- | --- | --- | --- | --- |
| Element | Line Type | Apparent Concentration | k Ratio | Wt% | Wt% Sigma | Standard Label | Factory Standard | Standard Calibration Date |
| C | K series | 1.77 | 0.01772 | 9.42 | 1.53 | C Vit | Yes |  |
| O | K series | 49.83 | 0.16770 | 45.65 | 0.92 | SiO2 | Yes |  |
| Na | K series | 9.28 | 0.03917 | 7.92 | 0.25 | Albite | Yes |  |
| Mg | K series | 1.20 | 0.00797 | 1.35 | 0.12 | MgO | Yes |  |
| Al | K series | 9.18 | 0.06593 | 9.66 | 0.26 | Al2O3 | Yes |  |
| Si | K series | 11.75 | 0.09315 | 12.82 | 0.32 | SiO2 | Yes |  |
| P | K series | 1.40 | 0.00784 | 1.11 | 0.19 | GaP | Yes |  |
| S | K series | 1.25 | 0.01079 | 1.37 | 0.12 | FeS2 | Yes |  |
| Cl | K series | 0.29 | 0.00257 | 0.32 | 0.09 | NaCl | Yes |  |
| K | K series | 0.39 | 0.00329 | 0.38 | 0.10 | KBr | Yes |  |
| Ca | K series | 6.73 | 0.06012 | 6.78 | 0.23 | Wollastonite | Yes |  |
| Ti | K series | 0.21 | 0.00214 | 0.26 | 0.14 | Ti | Yes |  |
| Mn | K series | 0.00 | 0.00000 | 0.00 | 0.20 | Mn | Yes |  |
| Fe | K series | 2.39 | 0.02390 | 2.95 | 0.31 | Fe | Yes |  |
| Total: |  |  |  | 100.00 |  |  |  |  |
