# Supplementary material for: Properties characterization and microstructural analysis of alkali-activated solid waste-based materials with sawdust and wastewater integration
Source: PLoS One. 2025 Jan 3;20(1):e0313413. doi: 10.1371/journal.pone.0313413 (PMC11698524; doi:10.1371/journal.pone.0313413)
Supplement: S5 Fig — (ZIP) [file pone.0313413.s005.zip › S5_Fig/Fig 25 (up).docx]

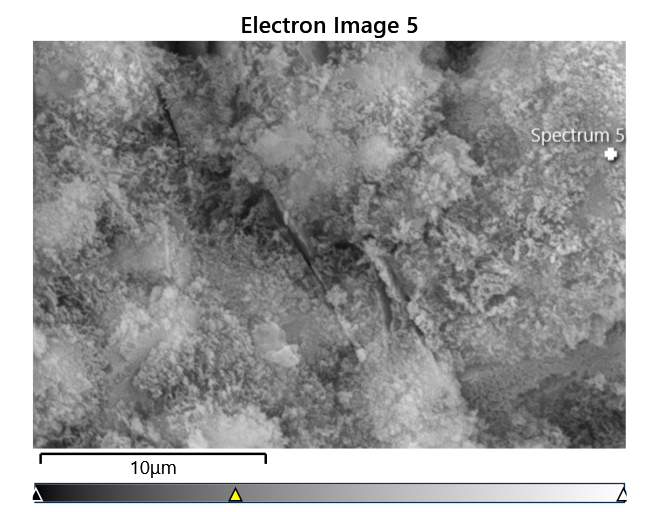

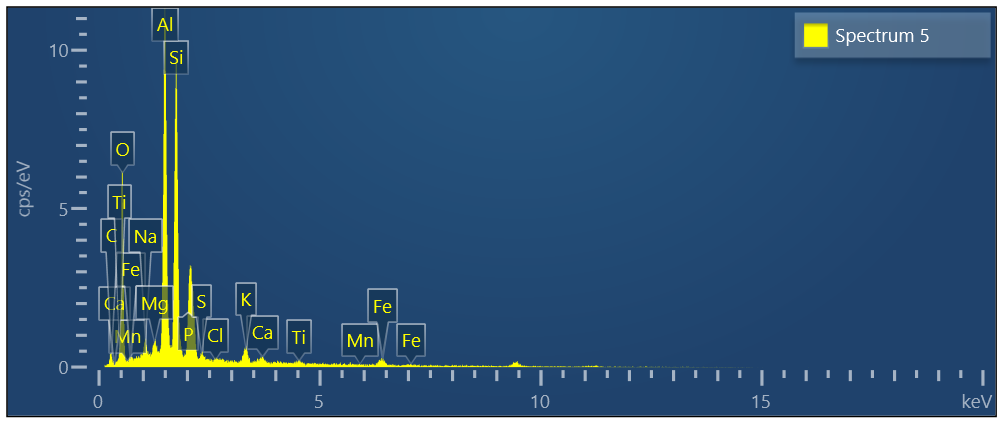


| **Spectrum 5** | | | | | | | | |
| --- | --- | --- | --- | --- | --- | --- | --- | --- |
| Element | Line Type | Apparent Concentration | k Ratio | Wt% | Wt% Sigma | Standard Label | Factory Standard | Standard Calibration Date |
| C | K series | 1.30 | 0.01296 | 11.20 | 1.99 | C Vit | Yes |  |
| O | K series | 29.55 | 0.09944 | 34.35 | 0.94 | SiO2 | Yes |  |
| Na | K series | 1.34 | 0.00566 | 1.24 | 0.14 | Albite | Yes |  |
| Mg | K series | 0.72 | 0.00478 | 0.83 | 0.12 | MgO | Yes |  |
| Al | K series | 18.36 | 0.13188 | 20.55 | 0.55 | Al2O3 | Yes |  |
| Si | K series | 17.02 | 0.13490 | 22.54 | 0.61 | SiO2 | Yes |  |
| P | K series | 1.32 | 0.00741 | 1.36 | 0.25 | GaP | Yes |  |
| S | K series | 0.60 | 0.00513 | 0.83 | 0.13 | FeS2 | Yes |  |
| Cl | K series | 0.11 | 0.00093 | 0.15 | 0.11 | NaCl | Yes |  |
| K | K series | 1.71 | 0.01446 | 2.07 | 0.16 | KBr | Yes |  |
| Ca | K series | 0.65 | 0.00579 | 0.80 | 0.14 | Wollastonite | Yes |  |
| Ti | K series | 0.43 | 0.00434 | 0.64 | 0.18 | Ti | Yes |  |
| Mn | K series | 0.06 | 0.00061 | 0.09 | 0.24 | Mn | Yes |  |
| Fe | K series | 2.32 | 0.02316 | 3.39 | 0.38 | Fe | Yes |  |
| Total: |  |  |  | 100.00 |  |  |  |  |
